# Suppression of Sin3A activity promotes differentiation of pluripotent cells into functional neurons

**DOI:** 10.1038/srep44818

**Published:** 2017-03-17

**Authors:** Debasish Halder, Chang-Hee Lee, Ji Young Hyun, Gyeong-Eon Chang, Eunji Cheong, Injae Shin

**Affiliations:** 1 National Creative Research Initiative Center for Biofunctional Molecules, Department of Chemistry, Yonsei University, Seoul 03722, Korea; 2 Department of Biotechnology, College of Life Science and Biotechnology, Yonsei University, Seoul 03722, Korea

## Abstract

Sin3 is a transcriptional corepressor for REST silencing machinery that represses multiple neuronal genes in non-neuronal cells. However, functions of Sin3 (Sin3A and Sin3B) in suppression of neuronal phenotypes are not well characterized. Herein we show that Sin3A knockdown impedes the repressive activity of REST and enhances differentiation of pluripotent P19 cells into electrophysiologically active neurons without inducing astrogenesis. It is also found that silencing Sin3B induces neurogenesis of P19 cells with a lower efficiency than Sin3A knockdown. The results suggest that Sin3A has a more profound effect on REST repressive machinery for silencing neuronal genes in P19 cells than Sin3B. Furthermore, we show that a peptide inhibitor of Sin3A-REST interactions promotes differentiation of P19 cells into functional neurons. Observations made in studies using genetic deletion and a synthetic inhibitor suggests that Sin3A plays an important role in the repression of neuronal genes by the REST regulatory mechanism.

Neuronal differentiation is a complicated, multistep process that relies on a network of various transcriptional factors and signaling molecules to regulate orderly acquisition and maintenance of neuronal traits. Normally, genes encoding neuronal phenotypic traits are poised or repressed in non-neuronal cells, neural progenitor cells and pluripotent stem cells. Upon receiving neurogenic environmental cues, these cells are differentiated into certain types of neurons. During past decades, many positive and negative transcriptional regulators have been identified as being critical for mediation of stage-specific neuronal gene expression and the sequential stages of neurogenesis.

The repressor element-1 (RE1) silencing transcription factor (REST, also called neuron-restrictive silencer element; NRSE) is known to be a representative master regulator that functions as a transcriptional repressor of multiple neuronal genes^1^,^2^. REST is a zinc finger protein that binds to RE1 sites residing in the promoters of many neuronal genes^1^,^3^. REST-mediated gene repression is achieved by the recruitment of two distinct corepressor complexes, Sin3 and CoREST complexes, at distinct domains (Supplementary Fig. S1)^4^,^5^,^6^,^7^. The N-terminal domain (1–152) of REST recruits a complex of Sin3 with histone deacetylase 1/2(HDAC 1/2) to suppress neuronal gene expression^4^,^6^. On the other hand, the C-terminal domain (525–1097) of REST interacts with the CoREST complex and exerts its repressor activity through silencing of genes essential for the neuronal phenotype^5^,^7^. It has been suggested that both N- and C-terminal domains of the REST complex are important for its repressive actions. The previous study shows that disruption of REST-CoREST interactions abrogates REST repressor activity and subsequently triggers differentiation of non-neuronal cells into neurogenic cells^5^. However, the role played by Sin3 in the REST repressive system, especially in the regulation of neuronal traits in neurons, is not well elucidated.

The mammalian Sin3 corepressor, which is linked to REST repressive machinery, governs the expression of neuronal genes through HDAC1/2 activities^8^,^9^. It is known that Sin3 does not directly bind to the RE1 site^10^ but associates with a transcription factor REST which binds to the RE1 region (a promoter site) of a number of genes encoding neuronal traits^1^,^3^. In the REST repressive machinery, Sin3 acts as a molecular adaptor or bridge through which various silencing factors or epigenetic modifiers are recruited to REST for its repressive function (Supplementary Fig. S1). Sin3 contains four paired amphipathic helix (PAH1-4) motifs. The N-terminal PAH1 and PAH2 motifs bind to REST^8^,^11^, and the region between PAH3 and PAH4 associates with almost all of the core components of the REST machinery, including HDAC1/2^8^,^12^. The two highly similar mammalian Sin3 proteins, Sin3A and Sin3B, have been shown to regulate overlapping but distinct biological functions^13^,^14^,^15^. Although many proteins are observed to interact with both of these isoforms^16^, others preferentially bind to one or the other^17^,^18^. To date, the functional roles played by Sin3A and Sin3B in mouse embryonic development and muscle differentiation have been investigated using a genetic deletion approach^14^,^19^. However, the functions of these two isoforms in regulating neuronal phenotypes remain largely unaddressed.

In this study, we have for the first time investigated the effect of suppression of Sin3A and Sin3B activities on neuronal differentiation of pluripotent cells. As described below, the results of knockdown studies show that silencing Sin3A abrogates the repressive activity of REST and enhances neuronal differentiation of pluripotent P19 cells to a higher degree than Sin3B knockdown. The findings suggest that Sin3A is a more important corepressor of REST functions that restrict neuronal phenotypes in pluripotent cells than Sin3B. Importantly, Sin3A knockdown promotes differentiation of P19 cells into electrophysiologically active neurons without generating astrocytes. In addition, we also show that a peptide inhibitor of binding of Sin3A to REST triggers differentiation of P19 cells into functional neurons but does not promote astrocyte differentiation.

## Results

### Effect of knockdown of Sin3A or Sin3B on neuronal differentiation of P19 cells

To examine whether suppression of Sin3A or Sin3B activity impedes the repressive function of REST and consequently enhances development of neuronal phenotypes, loss-of-function analyses were conducted in pluripotent P19 cells using Sin3A and Sin3B selective shRNAs. The results showed that Sin3A and Sin3B knockdown in P19 cells mediated by shRNAs blocked the expression of the respective Sin3A and Sin3B genes without affecting the expression of the other genes, indicating that selective and efficient repression of each endogenous gene takes place (Supplementary Fig. S2). The roles of Sin3A and Sin3B in the function of the REST silencing machinery were then assessed in Sin3A- and Sin3B-knockdown P19 cells. In this study, Sin3A- and Sin3B-knockdown P19 cells were allowed to aggregate for 3 days and aggregated embryonic bodies were then dissociated into single cells. Sin3A- and Sin3B-knockdown cells were cultured in monolayers for 10 days. The expression levels of REST target genes associated with stemness and neuronal differentiation were then measured by using quantitative RT-PCR analysis (Supplementary Table S1). Silencing of Sin3A in pluripotent P19 cells greatly affected the expression of REST target genes identified in previous studies^20^,^21^,^22^,^23^. Specifically, Sin3A-silenced cells expressed decreased levels of stemness factors, which are regulated by REST, and increased levels of neurogenesis-related REST target genes (Supplementary Fig. S3). However, Sin3B knockdown caused a smaller effect on the expression of these REST target genes in P19 cells than Sin3A silencing. The findings suggest that Sin3A might be a more active corepressor of REST silencing machinery than Sin3B. Specifically, Sin3A might likely be more involved in the repression of neuronal genes in pluripotent P19 cells than Sin3B.

**Figure 1:**
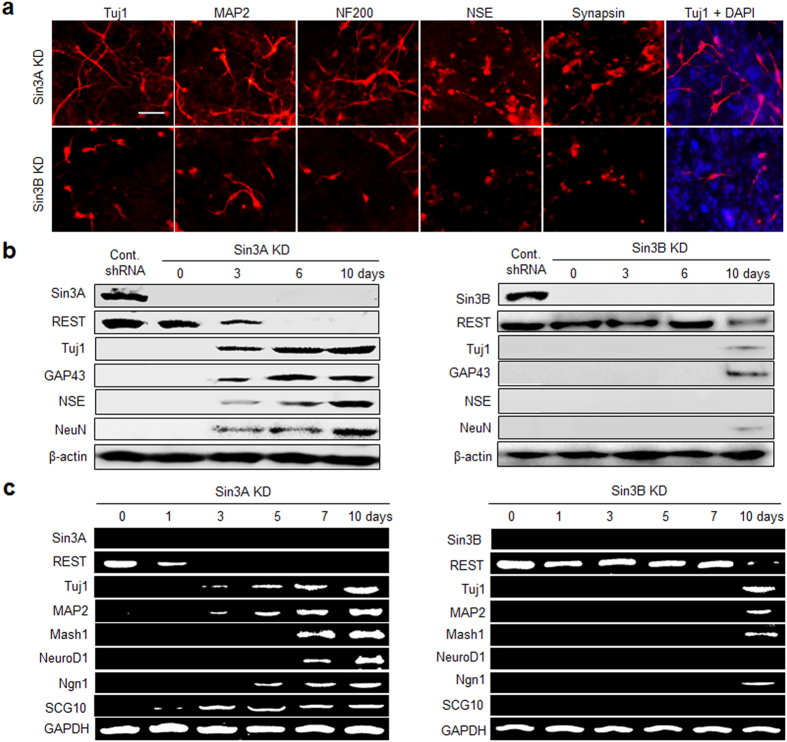
Effect of knockdown of Sin3A or Sin3B on the development of neuronal phenotypes in P19 cells. Sin3A or Sin3B-silenced P19 cells were allowed to aggregate in suspension for 3 days. The resulting embryoid bodies were cultured in a monolayer for 10 days. (**a**) The cells were immunostained with antibodies against neuronal markers; neuron-specific class III β-tubulin (Tuj1), microtubule-associated protein 2 (MAP2), neurofilament 200 (NF200), neuron-specific enolase (NSE), and synapsin. Scale bar, 50 μm. (**b**) Expression levels of the indicated neuronal marker genes were examined using western blot and (**c**) RT-PCR analyses. Scrambled shRNA (control shRNA) was used as a negative control. β-Actin and GAPDH were used as a loading control. All experiments were conducted at least three times.

To further explore the role of Sin3A and Sin3B in the suppression of neurogenesis, the effect of Sin3A and Sin3B knockdown on neuronal differentiation was evaluated by examining the expression of neuronal markers in cells. For this purpose, Sin3A- and Sin3B-knockdown P19 cells were cultured in monolayers for 10 days and then subjected to immunochemical analysis using several antibodies against neuron-specific markers (Supplementary Table S2). Sin3A-silenced P19 cells were differentiated into neurogenic cells with a higher efficiency than Sin3B knockdown cells (Fig. 1a). Neuronal differentiation of Sin3A- and Sin3B-silenced cells was also examined using western blot (Supplementary Table S3) and RT-PCR analyses (Supplementary Table S1) of neuronal markers. Whereas neuronal markers were highly expressed in Sin3A-silenced cells, very little or no expression of these markers occurred in Sin3B-silenced cells (Fig. 1b and c). It was also found that silencing Sin3A led to a decrease in expression of REST concomitant with an increase in expression of neuronal markers. However, Sin3B knockdown had only a small effect on the expression of REST and neuronal genes. Because REST expression is generally high in non-neuronal and pluripotent stem cells and is attenuated with the onset of neural differentiation^24^, its downregulation appears to be essential for de-repression of neuronal traits and acceleration of the neuronal phenotype in P19 cells. The findings again support the notion that Sin3A is likely to be more associated with REST-mediated restriction of neuronal traits to neurons in pluripotent P19 cells than Sin3B.

Further support for the above findings came from a comparative transcriptome analysis using mouse DNA chips. The results of gene expression profiling showed that stemness-related REST target genes^20^,^21^,^22^,^23^ were more downregulated in the Sin3A-knockdown P19 cells than in Sin3B-knockdown cells (Supplementary Table S4). In addition, neurogenesis-associated REST target genes^20^,^21^,^22^,^23^ were more upregulated in the Sin3A-knockdown cells than in the Sin3B-knockdown cells (Supplementary Table S4). Notably, upregulation of genes associated with neurogenesis, neurotransmitter receptors, synaptic vesicle mediated transmission/axonogenesis, and ion channels was more distinctive in Sin3A-silenced cells than in Sin3B-knockdown cells (Supplementary Table S5). Collectively, the findings suggest that ablation of Sin3A activity has a greater effect on differentiation of pluripotent P19 cells into neuronal cells than does abrogation of Sin3B activity. As a result, Sin3A is likely a more important corepressor for the repressive function of REST to suppress neuronal differentiation of pluripotent cells than Sin3B.

**Figure 2:**
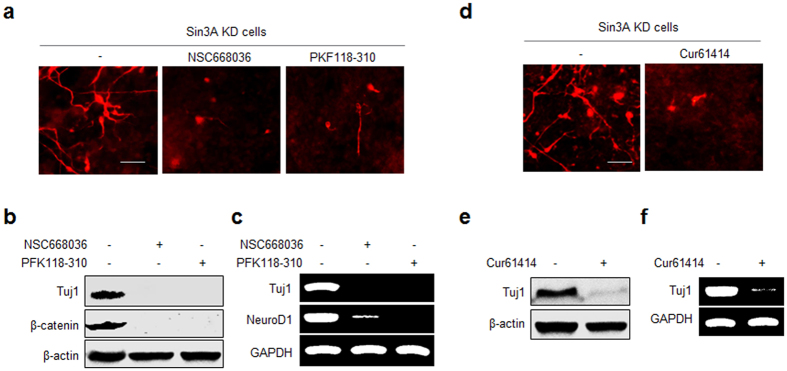
Signaling pathways associated with Sin3A knockdown-induced neurogenesis in P19 cells. (**a**–**c**) Sin3A knockdown P19 cells were incubated for 10 days in the absence and presence of an inhibitor for the Wnt pathway, 30 μM NSC668036 or 25 nM PFK118-310. (**a**) The cells were immunostained with Tuj1 antibody (bar: 50 mm). (**b**) Expression levels of β-catenin and Tuj1 were examined by using western blot analysis. (**c**) Transcriptional levels of Tuj1 and NeuroD1 were examined by using RT-PCR. (**d-e**) Sin3A knockdown P19 cells were incubated for 10 days in the absence and presence of an inhibitor for the Shh pathway, 30 μM Cur61414. (**d**) The cells were immunostained with Tuj1 antibody (bar: 50 mm). (**e**) The expression level of Tuj1 was examined by using western blot and (**f**) RT-PCR analyses. All experiments were conducted at least three times.

Observations made in previous studies show that the Wnt/β-catenin and Shh signaling pathways are associated with REST-mediated function in nervous system development^20^,^25^,^26^. In addition, the earlier effort demonstrated that Sin3A activity is regulated through the Wnt/β-catenin signaling pathway in Drosophila development^27^. On this basis, we tested whether Wnt/β-catenin and Shh signaling pathways were involved in neuronal differentiation induced by Sin3A knockdown in pluripotent P19 cells. Initially, Sin3A-knockdown P19 cells were cultured in the presence and absence of inhibitors (NSC668036 and PKF118-310) of the Wnt/β-catenin pathway. NSC668036 is an upstream inhibitor for the Wnt/β-catenin pathway that binds to the PDZ domain of Dishevelled (Dvl/Dsh) protein, a key component of Wnt signaling, and inhibits its interaction with Frizzled (Fzd)^28^. PKF118-310 is a downstream inhibitor for the Wnt/β-catenin pathway that blocks the formation of the complex between Tcf4 and β-catenin. The results showed that the neurogenesis inducing capability of Sin3A-silenced cells was attenuated in the presence of each of these inhibitors compared to that of untreated cells (Fig. 2a–c). In addition, the translational level of β-catenin in cells treated with each inhibitor was markedly decreased compared to that in cells untreated with an inhibitor (Fig. 2b). Moreover, the transcriptional level of NeuroD1, a Wnt/β-catenin-pathway target gene^29^, was also greatly reduced in cells treated with an inhibitor (Fig. 2c). These findings suggest that the Wnt/β-catenin signaling pathway is likely responsible for the neuronal fate commitment of Sin3A-silenced P19 cells.

Next, Sin3A-silenced P19 cells were cultured in the presence and absence of Cur61414, a known inhibitor of smoothened (SMO) in the Shh signaling pathway^30^. Observations made in immunocytochemistry, western blot and RT-PCR analyses indicate that the neurogenesis-inducing ability of Sin3A-knockdown cells is greatly reduced in the presence of the Shh signaling inhibitor (Fig. 2d–f).

The results of transcriptome analysis also revealed that a number of REST target genes associated with the Wnt/β-catenin and Shh signaling pathways^20^,^26^ were upregulated in Sin3A-silenced cells compared to control cells (Supplementary Table S6). In addition, Sin3A silencing promoted upregulation of many genes in P19 cells related to the Wnt/β-catenin and Shh pathways as well as genes coding for negative cell cycle regulators (Pitx2, Ccnd2 and Macf1)^31^. Furthermore, transcription regulators such as Apc2, Wif1, Sfrp5 and Zic2, which suppress the expression of β-catenin, were downregulated in Sin3A-silenced cells. However, RSPO2, the potent activator of β-catenin, was upregulated in these silenced cells^32^. Moreover, ingenuity pathway analysis (IPA) of the DNA chip data showed that several genes, which undergo a more than two-fold change after knockdown of Sin3A in P19 cells, were crosslinked with the Wnt/β-catenin and Shh signaling pathways (Supplementary Fig. S4). Taken together, the findings suggest that Wnt/β-catenin and Shh signaling pathways could be involved in neuronal differentiation of pluripotent P19 cells induced by suppression of Sin3A activity.

### Sin3A- and Sin3B-silenced P19 cells are not differentiated into astrocytes

**Figure 3:**
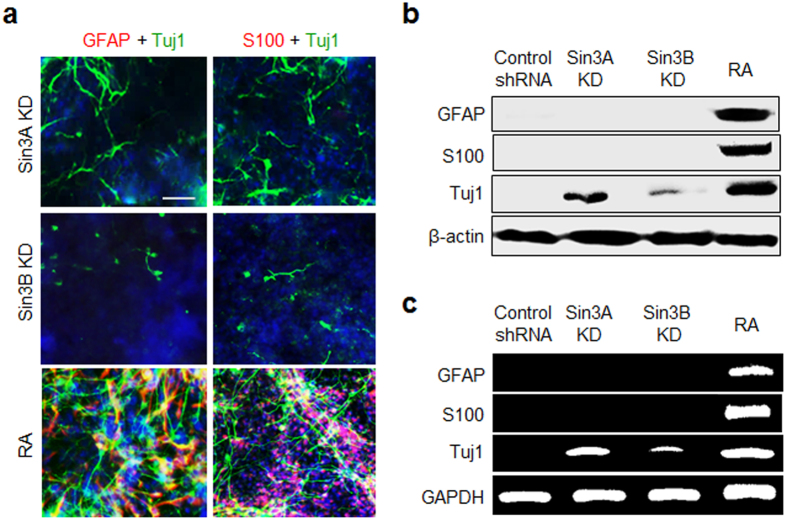
Sin3A- and Sin3B-silenced P19 cells are not converted to astrocytes. Sin3A- and Sin3B-knockdown P19 cells were allowed to aggregate in suspension for 3 days. The resulting embryoid bodies were cultured in a monolayer for 10 days. As a control, untransfected P19 cells were incubated with 1 μM retinoic acid (RA) for 10 days. (**a**) The cells were immunostained with antibodies against neuronal (Tuj1, green) and astrocyte markers (GFAP and S100, red). The nucleus of the cells was stained with DAPI (blue). Scale bar, 50 μm. (**b**) Expression levels of astrocyte markers in the Sin3A- and Sin3B-knockdown P19 cells were examined using western blot and (**c**) RT-PCR analyses. Scrambled shRNA (control shRNA) was used as a negative control. All experiments were conducted at least three times.

The results of a previous study indicate that REST-silenced embryonic and neural stem cells have the capacity to differentiate into astrocytes^20^. Because Sin3 serves as an important factor for the repressive function of REST, we examined whether Sin3A- or Sin3B-silenced P19 cells were also differentiated into astrocytes. In this study, after 3 days of aggregate formation in suspension, Sin3A- and Sin3B-knockdown cells were grown in monolayer culture for 10 days. As a control, untransfected P19 cells were incubated for 10 days with retinoic acid (RA), which is known to promote both neurogenesis and astrogenesis^33^. The expression levels of astrocyte markers in the cells, such as glial fibrillary acidic protein (GFAP) and S100, were then examined. The results of the immunocytochemical analysis showed that Sin3A-silenced cells were differentiated into neurons without the generation of astrocytes, as inferred from observations of positive staining by the Tuj1 antibody and negative staining by the GFAP and S100 antibodies (Fig. 3a). Also, Sin3B-silenced cells were not converted into astrocytes but were differentiated into neurons with a lower efficiency than Sin3A-knockdown cells (Fig. 3a). As expected, RA-treated P19 cells underwent neurogenesis and astrogenesis (Fig. 3a).

These observations gained support from the results of western blot and RT-PCR analyses of astrocyte markers, which showed that, unlike cells treated with RA, Sin3A- and Sin3B-silenced cells did not express astrocyte-specific genes (Fig. 3b,c). The gene expression profiling results also revealed that expression levels of astrogenesis-related genes did not change significantly in Sin3A- or Sin3B-knockdown P19 cells (Supplementary Table S7). Collectively, the observations made in this phenotype study indicate that suppression of Sin3A and Sin3B activities does not promote astrocyte differentiation in P19 cells. Rather, repression of Sin3A and Sin3B genes in pluripotent P19 cells induces neuronal differentiation with different levels of efficiency.

### Functionally active neurons are generated by knockdown of Sin3A in P19 cells

As described above, Sin3A has a more profound effect on REST repressive machinery for silencing neuronal genes in P19 cells than Sin3B and suppression of Sin3A activity induces neurogenesis of the cells to a greater degree than that of Sin3B activity. The findings led us to determine whether Sin3A knockdown P19 cells are differentiated into functional neurons. Voltage-gated sodium ion channels are a class of transmembrane proteins that are responsible for the rising phase of action potentials in excitable neuronal cells. Analysis of DNA chip data shows that sodium ion channel genes are upregulated in Sin3A-silenced P19 cells (Supplementary Table S5). To confirm that voltage-gated sodium ion channels are expressed, Sin3A-knockdown P19 cells were subjected to RT-PCR analysis. Indeed, expression levels of several sodium ion channels were found to increase in Sin3A-silenced cells (Supplementary Fig. S5).

**Figure 4:**
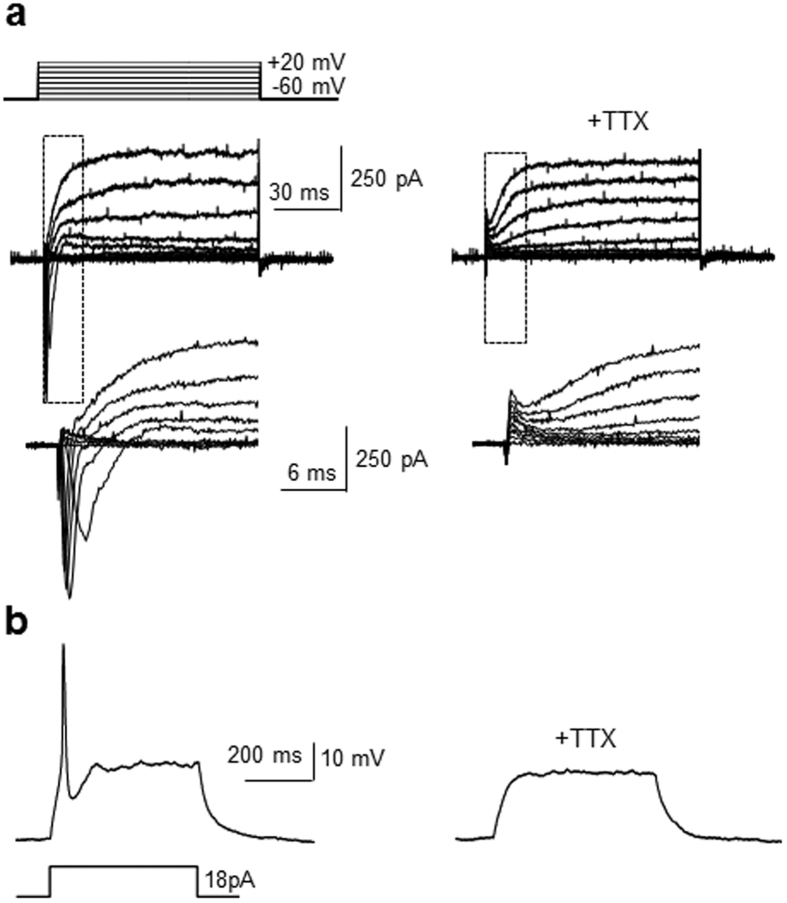
Sin3A-silenced P19 cells are differentiated into electrophysiologically active neurons. Sin3A-knockdown P19 cells were incubated for 21 days. (**a**) Whole-cell patch clamp recording of differentiated cells reveals generation of fast activating currents induced by 10 mV depolarizing voltage steps from −60 mV to + 20 mV before (left) and after (right) 0.5 μM tetrodotoxin (TTX) perfusion (n = 4/7 of recorded cells). Insets show respective traces on an expanded scale. (**b**) Action potentials generated by current injection in the differentiated cells (left) before and (right) after 0.5 μM TTX perfusion (n = 4/7 of recorded cells).

Because Sin3A knockdown led to upregulation of voltage-gated sodium ion channels, the electrophysiological properties of neurons differentiated from Sin3A-silenced P19 cells were determined using the whole-cells patch clamp technique. Sin3A-knockdown P19 cells were incubated for *ca.* 3 weeks and the differentiated cells were then subjected to patch-clamp analysis. Whole-cell currents elicited by 10 mV depolarizing voltage steps from −60 mV to +10 mV were recorded in the voltage–clamp mode. Approximately 60% of recorded cells were found to generate voltage-dependent, fast-inactivating inward and outward currents (Fig. 4a). However, when the selective blocker of sodium channels, tetrodoxin (TTX)^33^, was present, step depolarization-induced currents were not observed in the differentiated cells. These findings indicate that the current observed in these cells is generated through sodium ion channels.

The neuron-like cells, arising by Sin3A silencing in P19 cells, were also subjected to a current pulse in order to determine whether they are capable of generating depolarization-induced action potentials. The results of these measurements showed that the cells generated action potentials of relatively high amplitude in response to current injections (Fig. 4b). However, treatment with TTX almost completely blocked action potentials elicited by current injections. These results also indicate that the recorded action potentials in the cells are sodium channel*-*dependent. Collectively, the findings serve as evidence to support the conclusion that Sin3A silencing enhances differentiation of pluripotent P19 cells into neurons that have electrophysiological activity.

### Mad1 peptide blocks binding of Sin3A to REST

The results of gene knockdown studies suggest that substances that suppress Sin3A activity by blocking its interactions with REST could induce neuronal differentiation of P19 cells. If this proposal is correct, a synthetic inhibitor of Sin3A-REST interactions should promote neurogenesis of these cells. Previous studies have shown that the N-terminal PAH1 and PAH2 domains of Sin3A bind to the N-terminal region of REST^6^. Sequence 6–21 (RMNIQMLLEAADYLER, called Mad1 peptide) of the Mad1 protein is known to bind to Sin3A PAH2 with *K*_d_ = 29 nM^34^, and the sequence 171–186 (AEMIALAGLLQMSQGE, called SAP25 peptide) of the SAP25 protein to Sin3A PAH1 with *K*_d_ = 134 nM^35^. However, each of these peptides only very weakly associates with the opposite peptide. On this basis, we examined the inhibitory effect of Mad1 and SAP25 peptides on Sin3A-REST interactions.

The peptides used in this study were synthesized on a solid support employing a conventional Fmoc/t-Bu strategy (Supplementary Table S8)^36^. Binding of the Mad1 and SAP25 peptides to mouse Sin3A PAH1 (residues 114–195) and PAH2 (residues 295–385) was determined using fluorescence polarization. FITC-labeled Mad1 and SAP25 peptides were incubated separately with various concentrations of PAH1 and PAH2. The SAP25 peptide was found to bind PAH1 much more tightly than PAH2, and the Mad1 peptide was observed to interact with PAH2 much more strongly than PAH1 (Supplementary Fig. S6). We also examined binding properties of Sin3A PAH1 and PAH2 to mouse REST (residues 31–120) by using an ELISA. The results reveal that the PAH1 and PAH2 domains bind with near equal strengths to REST (Supplementary Fig. S7).

**Figure 5:**
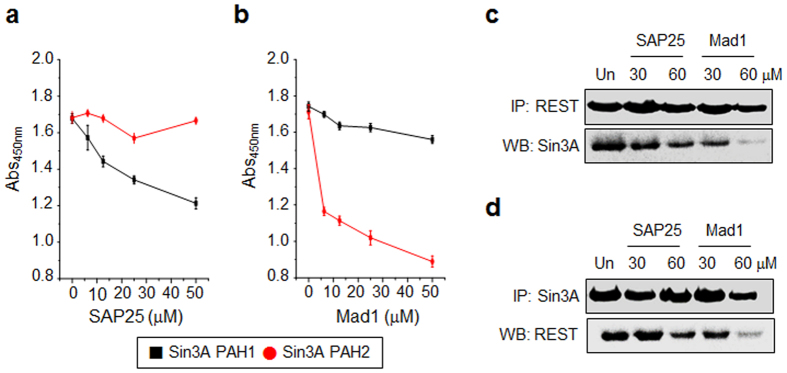
Inhibitory effect of the SAP25 and Mad1 peptides on binding of Sin3A PAH1 or PAH2 to REST. Mouse Sin3A PAH1 or PAH2 (1 μg/mL) coated wells were pre-incubated with various concentrations of (**a**) SAP25 or (**b**) Mad1 peptide. After 2 h, the mixtures were treated with GST-REST (2 μg/mL) for 2 h. Binding of GST-REST to PAH1 and PAH2 was measured using the anti-GST antibody and HRP conjugated secondary antibody (mean ± s.d., n = 3). (**c**-**d**) P19 cell lysates were incubated with indicated concentrations of the SAP25 or MAD1 peptide for 2 h. Immunoprecipitation was performed with antibodies against (**c**) REST and (**d**) Sin3A, and the amount of co-precipitated proteins was then determined using western blot analysis. ‘Un’ indicates no treatment with a peptide. All experiments were conducted at least three times.

We next examined whether the Mad1 and SAP25 peptides block the Sin3A-REST interaction. First, the ability of these peptides to inhibit the associations of PAH1 and PAH2 with REST was determined using ELISA. The results showed that the level of inhibition of binding of PAH2 to REST by Mad1 was greater than that of SAP25 toward PAH1-REST interactions (Fig. 5a,b). However, the Mad1 and SAP25 peptides had little effect on the respective PAH1-REST and PAH2-REST interactions. The cellular inhibitory activities of these peptides were then determined using an immunoprecipitation analysis. After incubating P19 cell lysates with several concentrations of SAP25 and MAD1 peptides, REST in the cell lysates was immunoprecipitated using the anti-REST antibody. The precipitates were probed using the anti-Sin3A antibody. Conversely, Sin3A in cell lysates was precipitated utilizing the anti-Sin3A antibody and the precipitates were immunoblotted with the anti-REST antibody. The results showed that the Mad1 peptide exhibited a much greater ability to block interactions of Sin3A with REST than the SAP25 peptide (Fig. 5c,d).

### TAT-NLS-Mad1 peptide induces differentiation of P19 cells into functional neurons

**Figure 6:**
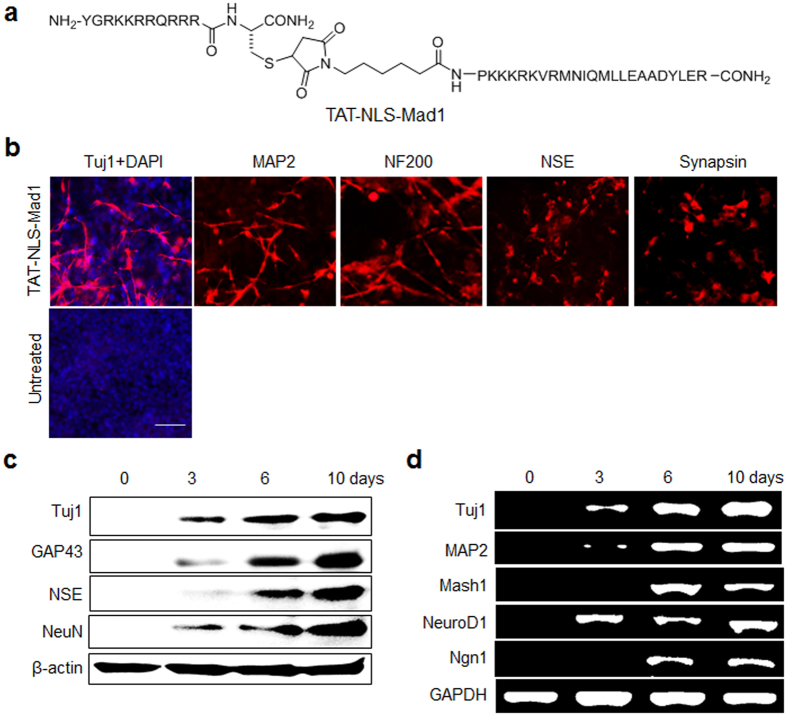
TAT-NLS-Mad1 peptide accelerates neuronal differentiation of P19 cells. (**a**) Structure of TAT-NLS-Mad1. (**b**) P19 cells were grown in suspension in the presence or absence of 30 μM TAT-NLS-Mad1 for a span of 3 days followed by culture in monolayers for 10 days. The cells were immunostained with antibodies against neuronal markers (scale bar: 50 μm). (**c**) Expression levels of neuronal markers in peptide-treated P19 cells were examined using western blot and (**d**) RT-PCR analyses. All experiments were conducted at least three times.

The fact that the Mad1 peptide has the ability to block the interaction of Sin3A with REST in cells led us to investigate whether this peptide is capable of inducing differentiation of P19 cells into neurons. A major limitation of peptides in biological research is their poor cell plasma membrane and/or nuclear membrane permeability. To circumvent this problem, Mad1 peptide was fused at the N-terminus with the cell-penetrating TAT peptide (YGRKKRRQRRR) and a nucleus localization signal (NLS, PKKKRKV) (Fig. 6a)^33^,^37^. The engineered TAT-NLS-Mad1 peptide was prepared by using the sequence shown in supplementary Fig. S8. In addition, N-terminal FITC-labeled TAT-NLS-Mad1 peptide was synthesized in order to examine nuclear permeability. Following incubation with FITC-TAT-NLS-Mad1, P19 cells were subjected to confocal fluorescence microscopy image analysis. The cell nuclei exhibited green fluorescence, indicating that the TAT-NLS-Mad1 peptide enters the nucleus where Sin3A-REST interactions take place (Supplementary Fig. S10).

Because the TAT-NLS-Mad1 peptide has nuclear permeability, its effects on expression of REST target genes associated with stemness and neurogenesis were then examined using quantitative RT-PCR. Stemness-associated REST target genes were downregulated but neurogenesis-related REST target genes were upregulated following treatment with this peptide (Supplementary Fig. S11). These observations are consistent with those made in Sin3A-knockdown cells. To explore whether TAT-NLS-Mad1 has the ability to promote neuronal differentiation, P19 cells were incubated with the peptide for 10 days and then subjected to immunocytochemical, western blot and RT-PCR analyses. Notably, the TAT-NLS-Mad1 peptide promoted neuronal differentiation of P19 cells, as inferred from the increased expression levels of neuronal genes (Fig. 6b–d). The neurogenesis-inducing activity of the TAT-NLS-Mad1 peptide in P19 cells was found to be comparable to that induced by the Sin3A knockdown. However, TAT itself did not affect neuronal differentiation (Supplementary Fig. S12), indicating that neurogenesis inducing activity is caused by the action of the Mad1 peptide. Moreover, the TAT-NLS-Mad1 peptide did not induce differentiation of P19 cells into astrocytes, as demonstrated by the negative detection of astrocyte markers (GFAP and S100) in the treated cells (Supplementary Fig. S13). These findings indicate that the TAT-NLS-Mad1 peptide induces neuronal differentiation of pluripotent P19 cells without generating astrocytes, in a manner that is consistent with observations made in studies using Sin3A-silenced cells.

**Figure 7:**
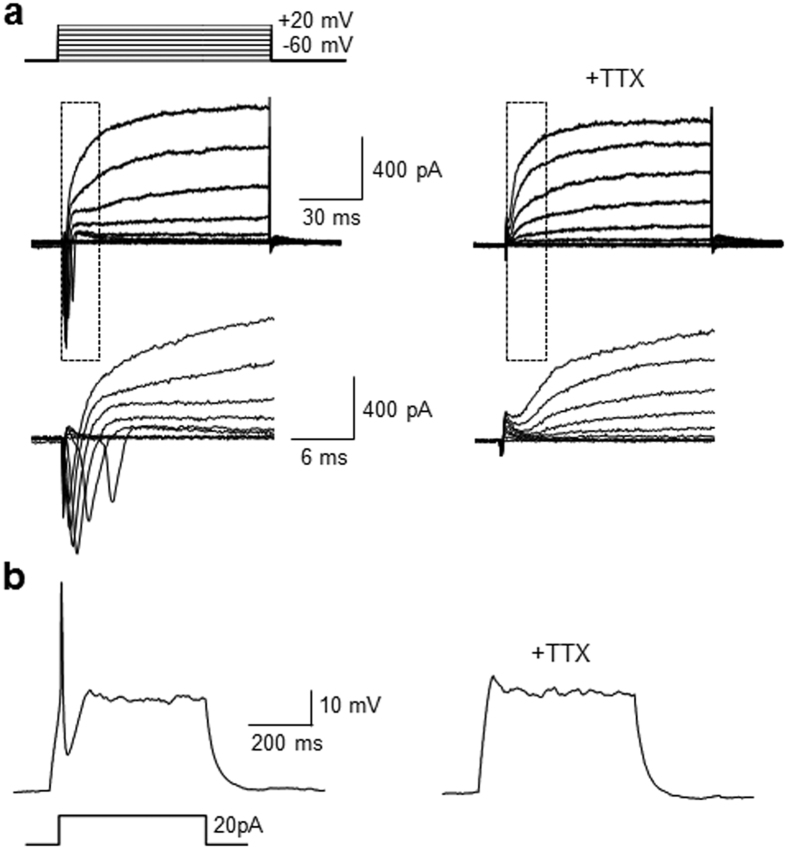
Neurogenic cells differentiated from P19 cells after treatment with TAT-NLS-Mad1 exhibit electrophysiological properties. P19 cells were incubated with 30 μM TAT-NLS-Mad1 for 21 days. (**a**) Representative traces of whole-cell current-clamp induced by 10 mV depolarizing voltage steps from −60 mV to +20 mV before (left) and after (right) 0.5 μM tetrodotoxin (TTX) perfusion (n = 5/12 of recorded cells). Insets show respective traces on an expanded scale. (**b**) Representative traces of an action potential recorded in differentiated Sin3A-knockdown cells in the current-clamp mode in response to depolarization by current injection before (left) and after (right) 0.5 μM TTX perfusion (n = 5/12 of recorded cells).

Finally, the electrophysiological properties of neurons differentiated from P19 cells treated with the TAT-NLS-Mad1 peptide were examined using whole cell patch-clamp analysis. The neuron-like cells formed in this manner were found to generate voltage-dependent, fast-inactivating inward and outward currents (Fig. 7a) as well as current injection induced action potentials with a relatively high amplitude (Fig. 7b). Furthermore, TTX almost completely abrogated the step depolarization-induced currents and current injection-promoted action potentials displayed by the differentiated neurons. These findings indicate that the currents and action potentials in the differentiated cells are sodium channel dependent. When taken together, the results of whole cell patch-clamp analysis showed that the TAT-NLS-Mad1 peptide accelerated differentiation of pluripotent P19 cells into electrophysiologically active neurons.

## Discussion

It is known that REST represses a number of neuronal genes by blocking their expression in non-neuronal and pluripotent/multipotent cells. Moreover, interactions of two distinct corepressors, Sin3 and CoREST, with the respective N- and C-terminal regions of REST are known to be crucial for REST-mediated gene repression^4^,^5^,^6^,^7^. Sin3A and REST transcripts have been shown to be coincidently distributed in mouse embryos, suggesting that Sin3A serves as a key constitutively required component for repression of genes by REST^38^. Although it has been shown that knockdown of CoREST leads to ablation of REST functions and to triggering differentiation of non-neuronal cells into neurogenic cells^5^, the roles of the mammalian Sin3 isoforms, Sin3A and Sin3B, in the suppression of neuronal phenotypes have not been well elucidated.

In the study described above, we have shown that Sin3A knockdown impedes REST repressive functions and promotes differentiation of pluripotent P19 cells into neurons. Silencing of Sin3A increases the expression of REST target genes, which are involved in the development of neuronal phenotypes, as well as neurogenesis-associated genes^20^,^21^. In contrast, Sin3A knockdown markedly reduces the expression of REST-related stemness genes, which are associated with the maintenance of pluripotency in stem cells. The results arising from this study indicate that silencing of Sin3A ablates pluripotency and induces differentiation of P19 cells into neurons. Notably, Sin3A-silenced pluripotent P19 cells differentiate into neurons without generating astrocytes. The results of the comparative transcriptome analysis indicate that Sin3A knockdown leads to an increase in expression of a number of genes regulated by REST that are important for neuronal physiology^20^,^21^. For instance, genes associated with neurite outgrowth, axonal guidance, vesicular transport, and neurotransmitter receptors and ion channels responsible for the generation of neuronal impulses are upregulated in the Sin3A-silenced P19 cells. Indeed, observations made in whole cell patch-clamp studies show that neuron-like cells differentiated from P19 cells by Sin3A silencing display the step depolarization-induced currents and current injection-promoted action potentials in a sodium channel-dependent manner. The findings serve as evidence to support the hypothesis that Sin3A knockdown leads to differentiation of pluripotent P19 cells into electrophysiologically active neurons.

Interestingly, the effect of Sin3B knockdown on de-repression of REST activity was found to be lower than that of Sin3A knockdown. In addition, silencing of Sin3B enhances neuronal differentiation of P19 cells less efficiently than does Sin3A knockdown. These findings suggest that Sin3A has a more profound effect on the suppression of neurogenesis by REST in pluripotent P19 cells than does Sin3B. Because Sin3A and Sin3B repress overlapping but distinct subsets of genes^15^,^17^,^18^, we surmised that the two isoforms might exhibit functional differences in repression of the neuronal genes that are exerted by the REST silencing system. When combined, the results suggest that Sin3A is a more essential corepressor for REST silencing machinery involved in the repression of neuronal genes in pluripotent P19 cells than Sin3B.

Uncovering substances that block Sin3A activity by inhibiting REST-Sin3A interactions and, consequently, trigger neuronal differentiation in P19 cells is of great interest. As a result, the finding that the Mad1 peptide composed of 16 amino acids has inhibitory activity against REST-Sin3A interactions in cells is highly significant. Importantly, the engineered TAT-NLS-Mad1 peptide promotes differentiation of P19 cells into neurons without producing astrocytes, phenomena that are observed in Sin3A-knockdown cells. In addition, neurons differentiated from pluripotent P19 cells treated with the TAT-NLS-Mad1 peptide generate sodium channel-dependent currents and action potentials, indicating that these neurons have electrophysiological activity.

Overall, the results of this effort suggest that the Sin3A corepressor plays a crucial role in the repression of neuronal genes by the REST regulatory mechanism. Thus, differentiation of pluripotent P19 cells into functionally active neurons can either be promoted by suppression of Sin3A activity by knockdown or an inhibitor of Sin3A-REST interactions. Finally, it is anticipated that the observations made in the present study will accelerate the understanding of the molecular mechanisms underlying REST-mediated repression of neuronal differentiation.

## Methods

### Cell culture

The P19 embryonic carcinoma cell line was purchased from the American Type Culture Collection (ATCC). Cells were grown in RPMI 1640 medium supplemented with 10% fetal bovine serum (FBS), 50 units/mL penicillin and 50 μg/mL streptomycin according to the procedure followed in previous studies^31^. The cells were grown to confluence in a humidified atmosphere (5% CO_2_) at 37 °C in 100 cm^2^ tissue culture dishes. The cells were sub-cultured after they formed a confluent monolayer (approximately 48 h).

### Neurogenesis of Sin3A and Sin3B knockdown P19 cells

Sin3A- and Sin3B-knockdown P19 cells were seeded at a density of 10^6^ cells/mL in 90 mm petri dishes under non-adherent culture conditions and allowed to aggregate for 3 days. Aggregated embryonic bodies were dissociated into single cells by treatment with 0.25% trypsin-EDTA solutions. The cells were then seeded in a tissue culture dish at a density of approximately 10^4^ cells/mL in culture media. After incubation for 24 h, culture media were replaced with low serum-containing media (RPMI 1640 supplemented with 4% FBS, 50 units/mL penicillin, 50 μg/mL streptomycin). The media were replenished every 2 days until cells were harvested on the indicative time.

### Electrophysiology

Whole-cell patch-clamp recordings were performed 21 days after differentiation induced by either Sin3A-silencing or peptide treatment of P19 cells. The cells with neuronal morphology (round cell body and neurite-like processes) were selected for whole-cell patch-clamp recordings. Cells plated on coverslips were placed in a submerged recording chamber (Warner instrument, Hadmen, CT, USA) on the microscope (Olympus, Japan). Electrophysiological recordings were made using a Multiclamp 700B amplifier (Molecular Devices, Foster City, CA, USA) and Clampex 10.3 of the pClamp software package (Molecular Devices). Digitization of voltages and currents were controlled by Digitizer 1440 A (Molecular Devices). Recording electrodes were made by pulling borosilicate capillary glass tubes (Warner Instruments) by using a pipet puller (P-97, Sutter Instrument, Novato, CA, USA). Pipette electrodes with a tip resistance ranging from 4 to 6 MΩ were filled with the intra-pipette solution (115 mM potassium gluconate, 10 mM KCl, 10 mM HEPES, 10 mM EGTA, 5 mM Mg^2+^-ATP and 0.5 mM 2Na^+^-GTP, pH 7.3 and 280–285 mOsm). All recordings were performed at room temperature and artificial cerebrospinal fluid (aCSF) was used as the external solution (124 mM NaCl, 3 mM KCl, 1.3 mM MgSO_4_, 1.25 mM NaH_2_PO_4_, 26 mM NaHCO_3_, 10 mM glucose and 2.4 mM CaCl_2_·2H_2_O). The external solution was aerated with O_2_ 95%/CO_2_ 5% mixed gas at room temperature. During the whole-cell configuration, the holding potential was clamped to −60 mV in the voltage clamp mode. To measure the sodium current, the cell was stimulated by 100 ms voltage steps of depolarizing current from the −60 mV holding the potential to +20 mV (+10 mV per step). In the current-clamp mode, the cells were subjected to a series of current injections to examine the generation of the action potential. Tetrodotoxin (0.5 μM TTX, Sigma-Aldrich) was applied to the external solution to confirm that the currents and action potentials are elicited by voltage-gated sodium channels. After perfusion with TTX in an external solution for 5–10 min, currents and action potentials were measured by using the above experimental process.

## Additional Information

**How to cite this article:** Halder, D. *et al*. Suppression of Sin3A activity promotes differentiation of pluripotent cells into functional neurons. *Sci. Rep.* **7**, 44818; doi: 10.1038/srep44818 (2017).

**Publisher's note:** Springer Nature remains neutral with regard to jurisdictional claims in published maps and institutional affiliations.

## Supplementary Material

Supplementary Information
