# Supplementary material for: Suppression of Sin3A activity promotes differentiation of pluripotent cells into functional neurons
Source: Sci Rep. 2017 Mar 17;7:44818. doi: 10.1038/srep44818 (PMC5356016; doi:10.1038/srep44818)
Supplement: Supplementary Information [file srep44818-s1.pdf]

Supplementary Information

Suppression of Sin3A activity promotes differentiation of pluripotent cells into functional neurons

Debasish Halder,[1] Chang-Hee Lee,[1] Ji Young Hyun,[1] Gyeong-Eon Chang,[2] Eunji Cheong[2] & Injae Shin[1,*]

[1]National Creative Research Initiative Center for Biofunctional Molecules, Department of Chemistry, Yonsei University, Seoul 03722, Korea

[2]Department of Biotechnology, College of Life Science and Biotechnology, Yonsei University, Seoul 03722, Korea

Supplementary Information

Construction of Sin3A and Sin3B knockdown P19 cells

Sin3A (sc-35974-SH), Sin3B (sc-35976-SH) and control (sc-108060) shRNA plasmids were purchased from Santa Crutz Biotechnology. Sin3A and Sin3B shRNA plasmids were a pool of 3 target-specific lentiviral vectors encoding 19-25 nt shRNAs designed to knock down gene expression, and contained a puromycin resistance gene for selection of stably transfected cells. The control shRNA plasmid encoded a scrambled shRNA sequence that did not degrade any known cellular mRNA. P19 cells were transfected with each plasmid according to the manufacturer's protocol. Transfected cells were selected with puromycin at a final concentration of 4 μg/mL for 4 weeks.

Reverse transcription polymerase chain reaction (RT-PCR)

Total RNAs were isolated using easy-BLUE (iNtRON Biotechnology, Inc.) from cells at different incubation periods. cDNA was prepared from total RNAs by using oligo (dT) primers and a Superscript II reverse transcriptase (Invitrogen) according to the manufacturer's instruction. The resulting cDNAs were used to analyze genes of interest by quantitative real time-PCR (qRT-PCR) and conventional RT-PCR with the Bio-Rad PCR system. The following PCR primers were purchased from Bioneer Company (Korea).

Table S1. Primers used for PCR

Experiment	Gene (Mouse)	Forward primer	Reverse primer	Amplicon size (bp)
**RT-PCR analysis**	Sin3A	5'- AGTGTCAACGTGGTCGAGAG-3'	5'- ATGCAGACGCTTCTTGCTTAC-3'	221
	Sin3B	5'- GAGGAGGTTTTCACAGAGGTG-3'	5'- GAGAACCCACACATGGACAAG-3'	240
	REST	5'- AGCCTTGCCAGTACGAAGC-3'	5'- CTTCAGGTGTGCCATGTAGTG-3'	224
	Tuj1	5'- CTCCCTTCGATTCCCTGGTC-3'	5'- TGCTCCGAGATGCGTTTGA-3'	439
	Map2	5'- GGATGGGCTTGTGTCTGATT-3'	5'- CTGGACCCACTCCACTCCACAAACT-3'	427
	NeuroD1	5'- GCATGCACGGGCTGAACGC-3'	5'- GGGATGCACCGGGAAGGAAG-3'	316
	Mash1	5'- CAAGTTGGTCAACCTGGGTT-3'	5'- GCTCTTGTTCCTCTGGGCTA-3'	293
	Ngn1	5'-CCAGCCACCACTTCAGTGTGATTT-3'	5'-TATTGTCAGCCGGCTCAAACCGAA-3'	417
	NF200	5'-AGCCTGCACTACTCGCTGA-3'	5'-GGCCGTTGCTTAGGGTGTC-3'	178
	SCG10	5'-CAGAGGAGCGAAGAAAGTCTCA-3'	5'-CTAGATTAGCCTCACGGTTTTCC-3'	180
	S100	5'-AATGTGTTCCATGCCCATTCG-3'	5'-ACCAGCACAACATACTCCTTG-3'	197
	GFAP	5'-TCTCGAATGACTCCTCCACTC-3'	5'-AAGCTCCGCCTGGTAGACAT-3'	243
	SCN1A	5'-TCAGAGGGAAGCACAGTAGAC-3'	5'-TTCCACGCTGATTTGACAGCA-3'	138
	SCN2A	5'-TTCATGGCTTCCAATCCCTCC-3'	5'-GGTGTCACGTCAGTCTTCTCT-3'	254
	SCN3A	5'-TTGCTGCTATCGAAAAGCGTG-3'	5'-GCACTGAATCGAAAAATTGCCT-3'	247
	SCN8A	5'-ACCCGTACTATTTGACGCAGA-3'	5'-TCCCTGTGAATGTGTACTCCA-3'	249
	GAPDH	5'-GCCATCAACGACCCCTTCAT-3'	5'-TTCACACCCATCACAAACAT	314
**Real time RT-PCR analysis**	Pou5f1 (Oct4)	5'CGGAAGAGAAAGCGAACTAGC-3'	5'ATTGGCGATGTGAGTGATCTG-3'	108
	Nanog	5'-AAAGGATGAAGTGCAAGCGGTGG-3'	5'-CTGGCTTTGCCCTGACTTTAAGC-3'	710
	Sox2	5'CGGCACAGATGCAACCGAT-3'	5'CCGTTCATGTAGGTCTGCG-3'	85
	Lin28a	5'-TGGTGTGTTCTGTATTGGGAGT-3'	5'-AGTTGTAGCACCTGTCTCCTTT-3'	86
	Snap25	5'-CCCACCACTACCATGGCCGAGGAC-3'	5'-CGGAATTCTTAACCACTTCCCAGCATC TT-3'	640
	Syn2	5'-CTTCAGCTCGCTGTCGCAA-3'	5'-CCACCAGGTTAAGCTCTGAGAAT-3'	215

	Gabrb3	5'-GGATGCTCACGGGAATATCCT-3'	5'-GCGGGACCACCTGTCTATG-3'	277
	Syt4	5'-CTCCTTGCACTTTACAGTTCTGA-3'	5'-TGGACTGATAACAGAGAGAGACC-3'	191
	Homer1	5'-CCCTCTCTCATGCTAGTTCAGC-3'	5'-GCACAGCGTTTGCTTGACT-3'	215
	Lepr	5'-GTCTTCGGGGATGTGAATGTC-3'	5'-ACCTAAGGGTGGATCGGGTTT-3'	154
	SCG10	5'-CAGAGGAGCGAAGAAAGTCTCA-3'	5'-CTAGATTAGCCTCACGGTTTTCC-3'	180
	Mbp	5'-TCACAGCGATCCAAGTACCTG-3'	5'-CCCCTGTCACCGCTAAAGAA-3'	125
	L1cam	5'-GGTTTCAGGGCATCTATCGCT-3'	5'-AGGCAGAACTACTGATTCTCCT-3'	152
	Nrxn3	5'-ACACCCAGGCGCAAATAGC-3'	5'-CTAGCCGGACACTTCCATTGA-3'	150
	Nefh	5'-AGCCTGCACTACTCGCTGA-3'	5'-GGCCGTTGCTTAGGGTGTC-3'	178
	Gria2	5'-TTTGGAGGTAGCCAACAGTTTC-3'	5'-AAAAATCGCATAGACGCCTCTT-3'	76

Immunocytochemistry

The differentiated P19 cells were fixed for 20 min with 4% paraformaldehyde and 0.1% Triton X-100 in PBS buffer. After washing with PBS, the fixed cells were incubated in a blocking solution (PBS containing 0.5% FBS) for 1 h at room temperature to reduce nonspecific binding of antibodies. The cells were treated with diluted primary antibodies in a blocking solution for 1 h at room temperature or overnight at 4 ^{o}C. After washing with PBS, the cells were treated with biotin-labeled secondary antibodies, followed by incubation with Cy3-conjugated streptavidin in a blocking solution for 1 h at room temperature. In some cases, Alexa Fluor® 488-conjugated goat anti-mouse IgG and goat anti-rabbit IgG were used as secondary antibodies. The cells were imaged by using a Nikon Eclipse TE2000 microscope.

Table S2. Antibodies and their concentrations used for immunocytochemistry

Name	Manufacturer	Clone/Host	Cat. No.	Dilution
Tuj1	Santa Cruz	Mouse monoclonal	sc-58888	1:200
NSE	Santa Cruz	Mouse monoclonal (clone NSE-P1)	sc-21738	1:200
NF200	Sigma Aldrich	Rabbit polyclonal	Sigma 4142	1:200
MAP2	Santa Cruz	Rabbit polyclonal (clone H-300)	sc-20172	1:200
Synapsin	Abcam	Rabbit polyclonal	ab64581	1:200
GFAP	Abcam	Rabbit polyclonal	ab7260	1:200
S100	Abcam	Rabbit polyclonal	ab868	1:100
Biotin-conjugated anti-mouse IgG	Sigma	Goat, Biotin-conjugated	B7264	1:200
Biotin-conjugated anti-rabbit IgG	Sigma	Goat, Biotin-conjugated	B7389	1:200
Alexa Fluor® 488 anti-mouse IgG	Life Technology	Goat, Fluorophore-conjugated	A11001	1:200
Alexa Fluor® 488 anti-rabbit IgG	Life Technology	Goat, Fluorophore-conjugated	A11034	1:200
Streptavidin-Cy3 Conjugate	Sigma	*Streptomyces avidinii*, Cy3-conjugated	S 6402	1:250

Western blot analysis

The differentiated P19 cells were washed with PBS twice and lysed with PRO-PREP™ Protein Extraction kit (iNtRON Biotechnology). Protein quantity was determined by using

SMART™ BCA Protein Assay Kit (iNtRON Biotechnology). Protein samples were separated by 8% or 12% polyacrylamide gels and transferred onto Immunobilon polyvinylidene difluoride (PVDF) membrane (Millipore) or nitrocellulose blotting membrane (Pall Corporation) in transfer buffer. The membranes were incubated in a blocking solution (TBS containing 5% non-fat skim milk and 0.5% Tween-20) for 1 h at room temperature to reduce nonspecific adsorption of antibodies. After washing with TBST (TBS buffer containing 0.5 % Tween-20), the membranes were incubated with diluted primary antibodies in TBST for 1 h at room temperature or overnight at 4 °C. After the membranes were washed with TBST, they were treated with diluted secondary antibodies in TBST for 1 h at room temperature. The treated membranes were visualized by using the ECL kit (Amersham Biotech).

Table S3. Antibodies and their concentrations used for western blot analysis>

Name	Manufacturer	Clone/Host	Cat. No.	Dilution
Sin3A	Abcam	Rabbit polyclonal (clone EPR6780)	ab129087	1:500
Sin3B	Santa Cruz	Mouse monoclonal (clone H-4)	sc-13145	1:500
REST	Santa Cruz	Rabbit polyclonal (clone H-290)	sc-25398	1:500
Tuj1	Santa Cruz	Mouse monoclonal	sc-58888	1:1000
Gap43	Santa Cruz	Mouse monoclonal (clone B-5)	sc-17790	1:1000
NSE	Santa Cruz	Mouse monoclonal (clone NSE-P1)	sc-21738	1:1000
NeuN	Millipore	Mouse monoclonal	MAB377	1:1000
GFAP	Abcam	Rabbit polyclonal	ab7260	1:1000
S100	Abcam	Rabbit polyclonal	ab868	1:500
β-catenin	Santa Cruz	Rabbit polyclonal (H-102)	sc-7199	1:1000
β-actin	Santa Cruz	Mouse monoclonal (clone C-4)	sc-47778	1:1000
anti-mouse IgG-HRP	Santa Crutz	Goat, HRP conjugated	Sc-2005	1:1000
anti-rabbit IgG-HRP	Santa Crutz	Goat, HRP conjugated	Sc-2004	1:1000

Signaling pathway study

Sin3A knockdown P19 cells were seeded at a density of 10^6 cells/mL in 90 mm petri dishes under non-adherent culture conditions and allowed to aggregate formation for 3 days. Aggregated embryonic bodies were dissociated into single cells by treatment with 0.25% trypsin-EDTA solution. The cells were seeded in a tissue culture dish at a density of approximately 2 x 10^5 cells/mL in culture media. After incubation for 24 h, the cells were treated for 10 days without or with an inhibitor for signaling pathways (an inhibitor for the Wnt pathway, 30 μM NSC668036 and 25 nM PKF118-310; an inhibitor for the Shh pathway, 30 μM Cur61414). Media containing each inhibitor were replenished every 2 days.

DNA microarray analysis

Sin3A and Sin3B knockdown P19 cells were incubated for 10 days without any treatment. mRNA samples were isolated using an mRNA extraction kit (iNtRON Biotechnology, Inc.) according to the manufacture's protocol. Samples were analyzed in duplicates on the GeneChip® Mouse Gene 2.0 ST Array (Affymetrix). To identify genes whose expression was changed substantially, Cy3-labeled RNA from differentiated Sin3A or Sin3B knockdown P19

cells were hybridized together with Cy5-labeled RNA from control P19 cells. Microarray data were analyzed with significance analysis of microarrays (p-value < 0.05). Genes exhibiting significant expression changes (>2-fold) were selected to investigate affected biological pathways.

Table S4. REST target genes that are affected by Sin3A and Sin3B knockdown in P19 cells

Classification	Gene Accession	Gene Symbol	Gene Description	M (Sin3A KD cells)	M (Sin3B KD cells)
Stemness	NM_011443	Sox2	SRY (sex determining region Y)-box 2	-1.210	-0.526
	NM_145833	Lin28a	lin-28 homolog A (C. elegans)	-1.025	-0.438
	NM_001252452	Pou5f1 (Oct4)	POU domain, class 5, transcription factor 1	-1.003	-0.297
	NM_001289828	Nanog	Nanog homeobox	0.443	1.447
	NM_001159500	Esrrb	estrogen related receptor, beta	-1.163	-0.545
Neuronal differentiation	NM_001291056	Snap25	synaptosomal-associated protein 25	2.087	0.222
	NM_001039195	Gria2	glutamate receptor, ionotropic, AMPA2 (alpha 2)	1.585	-0.246
	NM_001111015	Syn2	synapsin II	1.539	0.899
	NM_008553	Ascl1	achaete-scute complex homolog 1 (Drosophila)	3.181	-0.430
	ENSMUST00000124376	Gm16310	predicted gene 16310	1.989	0.789
	NM_001038701	Gabrb3	gamma-aminobutyric acid (GABA) A receptor, subunit beta 3	1.723	1.073
	NM_009308	Syt4	synaptotagmin IV	1.586	-0.136
	NM_001284189	Homer1	homer homolog 1 (Drosophila)	1.596	0.671
	NM_001122899	Lepr	leptin receptor	1.673	0.536
	NM_001110824	Foxp4	forkhead box P4	1.059	0.746
	NM_009867	Cdh4	cadherin 4	1.118	-0.078
	NM_001025251	Mbp	myelin basic protein	2.114	0.272
	NM_008478	L1cam	L1 cell adhesion molecule	1.328	0.347
	NM_010904	Nefh	neurofilament, heavy polypeptide	1.032	0.137
	NM_001198587	Nrxn3	neurexin III	1.099	0.005

M = Log_2 (fluorescent intensity of labeled Cy5 in mRNA obtained from Sin3A- or Sin3B-silenced P19 cells after 10 day incubation / (fluorescent intensity of labeled Cy3 in mRNA obtained from P19 cells transfected with the control shRNA).

Table S5. Neurogenesis related genes that are affected by Sin3A and Sin3B knockdown in P19 cells

Classification	Gene Accession	Gene Symbol	Gene Description	M (Sin3A-KD cells)	M (Sin3B-KD cells)
Neuronal differentiation	NM_001289916	Rora	RAR-related orphan receptor alpha	1.903	1.262
	NM_008927	Map2k1	mitogen-activated protein kinase kinase 1	1.086	0.710
	NM_016694	Park2	"Parkinson disease (autosomal recessive, juvenile) 2, parkin "	1.000	0.673
	NM_007447	Ang	"angiogenin, ribonuclease, RNase A family, 5 "	2.579	2.105
	NM_016721	Iqgap1	IQ motif containing GTPase activating protein 1	2.299	1.154
	ENSMUST00000018002	Ift52	intraflagellar transport 52	1.720	1.303
	NM_001081445	Ncam1	neural cell adhesion molecule 1	1.960	1.754
	NM_011682	Utrn	utrophin	1.746	1.094
	NM_177814	Erc2	ELKS/RAB6-interacting/CAST family member 2	1.179	0.939
	NM_008917	Ppt1	palmitoyl-protein thioesterase 1	1.144	0.205
	NM_025772	Dtnbp1	dystrobrevin binding protein 1	1.053	0.745
	NM_001195632	Arhgap32	Rho GTPase activating protein 32	1.033	0.909
	NM_001252192	Eya1	eyes absent 1 homolog (Drosophila)	1.944	1.241
	NM_001109757	Atp7a	"ATPase, Cu++ transporting, alpha polypeptide "	1.707	1.562
	NM_001109657	Gas7	growth arrest specific 7	1.370	0.758
	NM_153529	Nrn1	neuritin 1	1.064	0.858
	NM_009818	Ctnna1	"catenin (cadherin associated protein), alpha 1 "	1.958	1.184
	ENSMUST00000076654	Tes	testis derived transcript	1.213	0.639
	NM_010288	Gja1	"gap junction protein, alpha 1 "	1.610	0.666
	NM_008957	Ptch1	patched homolog 1	1.492	0.816
	NM_001114386	Nedd4l	"neural precursor cell expressed, developmentally down-regulated gene 4-like "	1.020	0.940
	NM_021310	Jmy	junction-mediating and regulatory protein	1.392	1.012
	NM_001004176	Maml3	mastermind like 3 (Drosophila)	1.153	0.688
	NM_001271768	Bhlhe41	"basic helix-loop-helix family, member e41 "	3.754	2.226
	NM_173440	Nrip1	nuclear receptor interacting protein 1	1.715	1.243
	NM_001168281	Wwtr1	WW domain containing transcription regulator 1	1.327	0.964
	NM_001136065	Hipk2	homeodomain interacting protein kinase 2	1.008	0.756
Cytoskeleton	NM_001165989	Ckap5	cytoskeleton associated protein 5	2.387	1.895
	NM_030238	Dync1h1	dynein cytoplasmic 1 heavy chain 1	2.113	1.921
	NM_001081363	Cenpf	centromere protein F	2.028	1.768
	NM_011682	Utrn	utrophin	1.746	1.692
	NM_001128606	Epb4.1	erythrocyte protein band 4.1	1.810	1.573
	NM_010864	Myo5a	myosin VA	1.837	1.698
	NM_001164099	Add3	adducin 3 (gamma)	1.950	1.763
	NM_010620	Kif15	kinesin family member 15	2.160	1.914
	NM_001081364	Arhgap21	Rho GTPase activating protein 21	2.487	1.698
	NM_007499	Atm	ataxia telangiectasia mutated	2.487	1.698
	NM_145148	Frmd4b	FERM domain containing 4B	2.487	1.698

	NM_177429	Ofd1	oral-facial-digital syndrome 1 gene homolog (human)	1.847	1.148
Cell adhesion molecules (CAMs)	NM_001081445	Ncam1	neural cell adhesion molecule 1	1.960	1.754
	NM_010578	Itgb1	integrin beta 1 (fibronectin receptor beta)	1.040	0.868
	NM_009864	Cdh1	cadherin 1	1.816	0.730
	NM_007664	Cdh2	cadherin 2	1.427	0.524
	NM_001004357	Cntnap2	contactin associated protein-like 2	1.254	0.297
	NM_010917	Nid1	nidogen 1	2.222	0.616
	NM_027514	Pvr	poliovirus receptor	1.852	0.355
	NM_053129	Pcdhb4	protocadherin beta 4	1.621	1.548
	NM_053133	Pcdhb8	protocadherin beta 8	2.146	1.524
	NM_001198823	App	amyloid beta (A4) precursor protein	1.552	1.293
	NM_001045489	Mfge8	milk fat globule-EGF factor 8 protein	1.716	1.289
	NM_026163	Pkp2	plakophilin 2	1.700	1.075
	NM_010581	Cd47	"CD47 antigen (Rh-related antigen, integrin-associated signal transducer) "	1.011	0.926
	NM_011581	Thbs2	thrombospondin 2	1.213	0.855
	NM_001199349	Cd99l2	CD99 antigen-like 2	1.422	0.808
	NM_001085508	Tmem8b	transmembrane protein 8B	1.027	0.772
Neurotransmitter receptor	NM_015730	Chrna4	"cholinergic receptor, nicotinic, alpha polypeptide 4 "	1.428	0.373
	NM_001024138	Gpr139	G protein-coupled receptor 139	1.270	0.906
	NM_008070	Gabrb2	gamma-aminobutyric acid (GABA) A receptor, subunit beta 2	1.623	0.179
	NM_020590	Gabarapl1	gamma-aminobutyric acid (GABA) A receptor-associated protein-like 1	1.095	0.897
	NM_001038701	Gabrb3	gamma-aminobutyric acid (GABA) A receptor, subunit beta 3	1.723	1.073
	NM_007974	F2rl1	coagulation factor II (thrombin) receptor-like 1	1.524	1.233
	NM_010169	F2r	coagulation factor II (thrombin) receptor	1.161	0.856
	NM_001042725	Calcr	calcitonin receptor	2.750	1.884
	NM_001177656	Grin1	"glutamate receptor, ionotropic, NMDA1 "	1.847	1.148
	NM_009602	Chrnb2	cholinergic receptor, nicotinic, beta polypeptide 2 (neuronal)	1.579	0.363
	NM_001111268	Grik2	glutamate receptor, ionotropic, kainate 2 (beta 2)	1.282	-0.240
	NM_001039195	Gria2	glutamate receptor, ionotropic, AMPA2 (alpha 2)	1.585	-0.246
	NM_001113325	Gria1	glutamate receptor, ionotropic, AMPA1 (alpha 1)	1.207	0.335
	NM_001012265	Olfr857	olfactory receptor 857	1.219	0.593
	NM_147012	Olfr1047	olfactory receptor 1047	1.147	0.871
	NM_147043	Olfr669	olfactory receptor 669	1.121	0.498
	NM_146711	Olfr43	olfactory receptor 43	1.092	0.666
	NM_009527	Wnt7a	"wingless-type MMTV integration site family, member 7A "	1.255	0.593
Ion channel activity	NM_001033336	Abcc4	"ATP-binding cassette, sub-family C (CFTR/MRP), member 4 "	1.546	1.147
	NM_001271873	Ano10	anoctamin 10	1.453	0.958
	NM_172621	Clic5	chloride intracellular channel 5	1.238	0.865
	NM_145947	Slc26a7	"solute carrier family 26, member 7 "	1.060	0.580

	ENSMUST0000 0029932	Clca1	chloride channel calcium activated 1	1.051	0.611
	NM_145947	Slc26a7	"solute carrier family 26, member 7 "	1.060	0.580
	NM_013491	Clcn1	chloride channel 1	1.245	1.062
	NM_018733	Scn1a	"sodium channel, voltage-gated, type I, alpha "	1.034	0.270
	NM_001099298	Scn2a1	"sodium channel, voltage-gated, type II, alpha 1 "	1.208	1.069
	NM_018732	Scn3a	"sodium channel, voltage-gated, type III, alpha "	1.358	0.837
	NM_001077499	Scn8a	'sodium channel, voltage-gated, type VIII, alpha"	1.444	0.622
	ENSMUST0000 0056508	Clcn3	chloride channel 3	1.849	1.341
	NM_011929	Clcn6	chloride channel 6	1.268	1.386
	NM_152923	Kcnq3	"potassium voltage-gated channel, subfamily Q, member 3 "	1.547	1.110
	NM_023456	Npy	neuropeptide Y	1.445	0.851
	NM_009162	Scg5	secretogranin V	1.349	1.274
Neurite outgrowth/ axonogenesis	NM_001290434	Epha7	Eph receptor A7	2.965	1.474
	NM_019413	Robo1	roundabout homolog 1 (Drosophila)	1.720	1.558
	NM_007936	Epha4	Eph receptor A4	2.238	0.937
	NM_145452	Rasa1	RAS p21 protein activator 1	1.262	0.794
	NM_001290753	Ephb2	Eph receptor B2	1.063	0.651
	NM_001146031	Nrcam	neuron-glia-CAM-related cell adhesion molecule	2.079	0.020
	NM_007960	Etv1	ets variant 1	1.110	0.730
	NM_018744	Sema6a	"sema domain, transmembrane domain (TM), and cytoplasmic domain, (semaphorin) 6A "	1.057	-0.138
	NM_001168296	Ephb1	Eph receptor B1	0.602	0.021
Synaptic vesicle transport	NM_001199304	Atxn1	ataxin 1	1.753	1.664
	NM_001159948	Als2	amyotrophic lateral sclerosis 2 (juvenile)	1.334	1.200
	NM_001291056	Snap25	synaptosomal-associated protein 25	2.087	0.222
	NM_009308	Syt4	synaptotagmin IV	1.586	-0.136
	NM_001029873	Unc13a	unc-13 homolog A (C. elegans)	1.714	0.757
	NM_001286033	Stx2	syntaxin 2	1.190	1.079
	NM_011026	P2rx4	"purinergic receptor P2X, ligand-gated ion channel 4 "	1.540	1.355
	ENSMUST000 00019268	Scrn1	secernin 1	2.019	0.624
	NM_008720	Npc1	Niemann-Pick type C1	1.984	1.442
	NM_172601	Rab2b	"RAB2B, member RAS oncogene family "	1.837	0.260
	NM_001045489	Mfge8	milk fat globule-EGF factor 8 protein	1.716	1.184
	NM_020505	Vav3	vav 3 oncogene	1.531	1.359
	NM_029153	Scamp1	secretory carrier membrane protein 1	1.452	1.269
	NM_130449	Colec12	collectin sub-family member 12	1.348	1.182
	NM_001114611	Stxbp5l	syntaxin binding protein 5-like	1.289	1.082
	NM_177333	Exoc3	exocyst complex component 3	1.073	0.880
	NM_010581	Cd47	"CD47 antigen (Rh-related antigen, integrin-associated signal transducer) "	1.011	0.926

	NM_001252520	Picalm	phosphatidylinositol binding clathrin assembly protein	1.055	0.857
Neurological system process	NM_010422	Hexb	hexosaminidase B	2.235	1.117
	ENSMUST00000076654	Tes	testis derived transcript	1.213	0.639
	NM_010299	Gm2a	GM2 ganglioside activator protein	1.447	1.255
	NM_009527	Wnt7a	"wingless-type MMTV integration site family, member 7A "	1.255	0.593
	NM_172621	Clic5	chloride intracellular channel 5	1.238	0.865
	NM_001173483	Rlbp1	retinaldehyde binding protein 1	1.185	0.860
	NM_025772	Dtnbp1	dystrobrevin binding protein 1	1.053	0.745
	NM_016694	Park2	"Parkinson disease (autosomal recessive, juvenile) 2, parkin "	1.000	0.673
	NM_001289575	Tsc1	tuberous sclerosis 1	1.158	1.042

M = Log_2 (fluorescent intensity of labeled Cy5 in mRNA obtained from Sin3A- or Sin3B-silenced P19 cells after 10 day incubation / (fluorescent intensity of labeled Cy3 in mRNA obtained from P19 cells transfected with the control shRNA).

Table S6. Signaling pathway associated genes that are affected by Sin3A knockdown in P19 cells

Classification	Gene Accession	Gene Symbol	Gene Description	M (Sin3A KD cells)
Wnt signaling pathway	NM_177993	Hbp1	high mobility group box transcription factor 1	2.321
	NM_021279	Wnt1	"wingless-type MMTV integration site family, member 1 "	1.287
	NM_011718	Wnt10b	"wingless-type MMTV integration site family, member 10B "	1.507
	NM_001025438	Camk2d	"calcium/calmodulin-dependent protein kinase II, delta "	1.494
	NM_001081146	Prickle2	prickle homolog 2 (Drosophila)	1.422
	NM_001081088	Lrp2	low density lipoprotein receptor-related protein 2	1.221
	NM_007501	Neurod4	neurogenic differentiation 4	1.503
	NM_201637	Chd8	chromodomain helicase DNA binding protein 8	0.855
	NM_010901	Nfatc3	"nuclear factor of activated T cells, cytoplasmic, calcineurin dependent 3 "	1.027
	NM_001164198	Prkacb	"protein kinase, cAMP dependent, catalytic, beta "	1.107
	NM_013834	Sfrp1	secreted frizzled-related protein 1	1.450
	NM_001081457	Ppp2r5c	"protein phosphatase 2, regulatory subunit B, gamma "	1.007
	NM_009527	Wnt7a	"wingless-type MMTV integration site family, member 7A "	1.255
	NM_001145959	Ndrg2	N-myc downstream regulated gene 2	2.814
	NM_021458	Fzd3	frizzled homolog 3 (Drosophila)	1.187
	ENSMUST00000109108	Wwox	WW domain-containing oxidoreductase	1.233
	NM_007395	Acvr1b	activin A receptor, type 1B	0.891
	NM_016979	Prkx	"protein kinase, X-linked "	1.390
	NM_009829	Ccnd2	cyclin D2	2.761
	NM_008514	Lrp6	low density lipoprotein receptor-related protein 6	1.176
	NM_001293622	Ppp3ca	"protein phosphatase 3, catalytic subunit, alpha isoform "	1.632
	NM_001199136	Macf1	microtubule-actin crosslinking factor 1	1.560
	NM_001042504	Pitx2	paired-like homeodomain transcription factor 2	1.057
	NM_172815	Rspo2	R-spondin 2 homolog (Xenopus laevis)	1.212
	XM_006511927	Ctnnb1	catenin (cadherin associated protein), beta 1	0.652
	NM_001031667	Gsk3a	glycogen synthase kinase 3 alpha	-1.128
	NM_011789	Apc2	adenomatosis polyposis coli 2	-0.661
	NM_011915	Wif1	Wnt inhibitory factor 1	-0.357
	AK051032	Zic2	zinc finger protein of the cerebellum 2	-1.681
	NM_016687	Sfrp5	secreted frizzled-related protein 5	-0.020
	NM_010091	Dvl1	"dishevelled, dsh homolog 1 (Drosophila) "	-0.974
Hedgehog signaling pathway	NM_008957	Ptch1	patched homolog 1	1.492
	NM_001081125	Gli2	GLI-Kruppel family member GLI2	1.319
	NM_173185	Csnk1g1	"casein kinase 1, gamma 1 "	1.239
	NM_021339	Cdon	cell adhesion molecule-related/down-regulated by oncogenes	1.738
	ENSMUST00000018002	Ift52	intraflagellar transport 52	1.720

	NM_001136065	Hipk2	homeodomain interacting protein kinase 2	1.626
	NM_026298	Ift172	intraflagellar transport 172	1.579
	NM_026110	Paxbp1	PAX3 and PAX7 binding protein 1	0.867
	NM_008781	Pax3	paired box 3	3.975
	NM_016979	Prkx	"protein kinase, X-linked "	1.390
	NM_001081088	Lrp2	low density lipoprotein receptor-related protein 2	1.221
	NM_144955	Nkx6-1	NK6 homeobox 1	1.005
	NM_010941	Nsdhl	NAD(P) dependent steroid dehydrogenase-like	1.120
	NM_001164198	Prkacb	"protein kinase, cAMP dependent, catalytic, beta "	1.107
	NM_021279	Wnt1	"wingless-type MMTV integration site family, member 1 "	1.287
	NM_011718	Wnt10b	"wingless-type MMTV integration site family, member 10B "	1.507
	NM_008130	Gli3	GLI-Kruppel family member GLI3	1.334

M = Log_2 (fluorescent intensity of labeled Cy5 in mRNA obtained from Sin3A-silenced P19 cells after 10 day incubation / (fluorescent intensity of labeled Cy3 in mRNA obtained from P19 cells transfected with the control shRNA). The genes in purple color are known as REST target genes.

Table S7. Astrogenesis related genes that are affected by Sin3A and Sin3B knockdown in P19 cells

Classification	Gene Accession	Gene Symbol	Gene Description	M (Sin3A KD cells)	M (Sin3B KD cells)
Astrogenesis related genes	NM_152915	Dner	delta/notch-like EGF-related receptor	-0.135	0.035
	NM_008052	Dtx1	deltex 1 homolog (Drosophila)	-0.009	-0.017
	NM_010153	Erbb3	v-erb-b2 erythroblastic leukemia viral oncogene homolog 3 (avian)	0.743	0.917
	NM_001277268	Fgf5	fibroblast growth factor 5	-1.201	-1.252
	NM_001131020	Gfap	glial fibrillary acidic protein	-0.392	-0.114
	BC063762	Gjb6	gap junction protein, beta 6	-0.446	-0.512
	NM_010814	Mog	myelin oligodendrocyte glycoprotein	-0.286	-0.585
	NM_008667	Nab1	Ngfi-A binding protein 1	0.095	0.412
	ENSMUST00000181702	Nrg1	neuregulin 1	-0.303	-0.572
	NM_011261	Reln	reelin	-0.142	-0.145
	NM_011437	Sox10	SRY (sex determining region Y)-box 10	-0.208	-0.121
	NM_011674	Ugt8a	UDP galactosyltransferase 8A	-0.557	-0.461
	NM_009115	S100b	S100 protein, beta polypeptide, neural	-0.034	-0.396
	NM_011309	S100a1	S100 calcium binding protein A1	0.208	0.495
	NM_145833	Lin28a	lin-28 homolog A (C. elegans)	-1.025	-0.438
	NM_011443	Sox2	SRY (sex determining region Y)-box 2	-1.210	-0.526
	NM_001252452	Pou5f1	"POU domain, class 5, transcription factor 1 "	-1.003	-0.297

M = Log_2 (fluorescent intensity of labeled Cy5 in mRNA obtained from Sin3A or Sin3B-silenced P19 cells after 10 day incubation / (fluorescent intensity of labeled Cy3 in mRNA obtained from P19 cells transfected with the control shRNA).

Preparation of recombinant vectors for expression of N-terminal His$_6$-tagged Sin3A PAH1 and PAH2 domains and C-terminal His$_6$-tagged GST-REST

BL21 (DE3) strain was used as a host for protein expression. cDNA clones of mouse Sin3A and mouse REST were purchased from 21C Human Gene Bank (Genome Research Center, KRIBB, Korea). Sin3A PAH1 and PAH2 domains and truncated REST were amplified by PCR using the following primers:

Sin3A PAH1 (114-195) (restriction enzyme: *NheI, XhoI*):
sense 5'-TATGGCTAGCGGACAGCAGCAGTTTCAGAGGCTC-3'
antisense 5'-GTGCTCGAGTTACTGCACCTCAATTTTGTAGCCAGGAG-3'
Sin3A PAH2 (295-385) (restriction enzyme: *NheI, XhoI*):
sense 5'-GCCATATGTCTTTGCAAAACA-3'
antisense 5'-CGCCTCGAGTTACTTCTCGAATT-3'
REST (31-120) (restriction enzyme: *BamHI, XhoI*):
sense 5'-TTGGATCCATGGACCTGCACGAGCTCTCGAAA - 3'
antisense 5'-ATCTCGAGTCAGTGGTGGTGGTGGTGGTGACCCTGGAAGTA CAAGTTCTCGGCTTCAAATACGGGCT-3'

Sin3A PAH1 and PAH2 genes were cloned into pET28a by using *Nhe*I/*Xho*I restriction enzymes. The REST gene was cloned into pGEX6p-1 by using *BamH*I/*Xho*I restriction enzymes.

Purification of His$_6$-tagged proteins

E. coli BL21 (DE3) competent cells were transformed with plasmids containing Sin3A PAH1, PAH2 or GST-REST gene. A single colony was grown at 37 ^{o}C for 14 h in 10 mL of Luria-Bertani (LB) media containing 50 μg/mL kanamycin for PAH1 and PAH2 or 50 μg/mL ampicillin for GST-REST with shaking. Protein expression was induced by incubation with 0.1 mM isopropyl-β-thiogalactopyranoside (IPTG, Bio Basic Canada Inc.) for 4 h at 37 ^{o}C. The cells were harvested by centrifugation (5,000 rpm, 10 min, 4 ^{o}C). The bacterial pellets were re-suspended with ice-cold binding buffer (20 mM Tris, 500 mM NaCl, 1 tablet of 3 protease inhibitor cocktail (Roche Applied Science, Mannheim, Germany), pH 7.6) and then lysed by sonication. Cell debris were removed by centrifugation (15,000 rpm, 30 min, 4 ^{o}C). The supernatant was filtered through 0.45 μm pore hydrophobic nitrocellulose membrane and the filtrate was poured on the Ni-NTA resin (Thermo) in column at 4 ^{o}C. After 30 min, the column was washed with 5 volumes of washing buffer (20 mM Tris 500mM NaCl, pH 7.6) five times. Proteins were eluted with 10 volumes of elution buffer (20 mM Tris 500mM NaCl, 200 mM imidazole, pH 7.6). The eluted protein solutions were dialyzed with Tris buffer (20 mM Tris, 150 mM NaCl, pH 7.6) and concentrated by Amicon Ultra centrifugal tube (Millipore, USA). Purified proteins were analyzed by using SDS-PAGE and visualized by Coomassie Blue staining.

Synthesis of peptides

Peptides used in this study were synthesized on a solid support by using conventional Fmoc/t-Bu strategy (Pai et al., 2012). TAT-NLS-Mad1 and FITC-TAT-NLS-Mad1 were prepared by

coupling YGRKKRRQRRRC or FITC-YGRKKRRQRRRC (1 equiv.) with N-terminal maleimide conjugated PKKKRKVRMNIQMLLEAADYLER (1.5 equiv.) in anhydrous DMSO in the presence of N-methylmorpholine (NMM, 3 equiv.) under argon atmosphere (Supplementary Fig. S8). After 4 h, the crude mixture was purified by using reversed-phase HPLC with a gradient of 5–100% CH_3CN (0.1% TFA) in water (0.1% TFA) over 60 min (Supplementary Fig. S9). The purified products were characterized by MALDI-TOF MS.

Table S8. Name and sequence of peptides used in this study and their MS data

Peptide name	Sequence	Molecular formula	Calculated mass $[M+H]^+$ or $[M+Na]^+$	Observed mass $[M+H]^+$ or $[M+Na]^+$
SAP25 peptide	AEMIALAGLLQMSQGE*X*	$C_{76}H_{133}N_{21}O_{24}S_2$	1788.9	1788.8
FITC-SAP25 peptide	FITC-AEMIALAGLLQMSQGE*X*	$C_{100}H_{148}N_{23}O_{30}S_3$	2248.0	2248.2
Mad1 peptide	RMNIQMLLEAADYLER*X*	$C_{90}H_{153}N_{27}O_{26}S_2$	2093.1	2093.0
FITC-Mad1 peptide	FITC-RMNIQMLLEAADYLER*X*	$C_{114}H_{168}N_{29}O_{32}S_3$	2552.1	2552.4
TAT-NLS-Mad1	YGRKKRRQRRRC-tether-PKKKRKVRMNIQMLLEAADYLER	$C_{201}H_{352}N_{74}O_{49}S_3$	4683.6	4683.3
FITC-TAT-NLS-Mad1	FITC-YGRKKRRQRRRC-tether-PKKKRKVRMNIQMLLEAADYLER	$C_{225}H_{368}N_{72}O_{55}S_4$	5143.7	5143.7

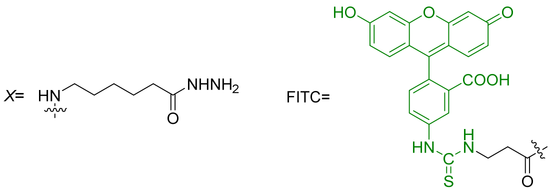

Fluorescence polarization assay

Various concentrations (0-10 μM) of PAH1 or PAH2 were mixed at room temperature with 1 μM FITC-SAP25 or FITC-Mad1 peptide in 20 mM Tris-HCl and 150 mM NaCl at pH 7.5 (a final volume: 20 μL) on non-coated black 384-well microplates (SPL Science). After 30 min, fluorescence polarization values were read on a microplate reader (Tecan Infinite F200, USA) at excitation and emission wavelengths of 495 and 520 nm. Fluorescence polarization values were expressed as mP units.

Enzyme-linked immunosorbent assay (ELISA)

Immunosorbant plates (96-well, Maxi-sorp, NUNC) were coated at 4 °C with 1 μg/mL PAH1, PAH2 or BSA in 20 mM sodium bicarbonate (pH 9.6) overnight. Protein coated-wells were washed with TBST solutions (20 mM Tris-HCl, 150 mM NaCl, 0.05% Tween 20, pH 7.5), blocked with 2.5% BSA in TBS for 2 h at room temperature. The blocked plates were washed with TBST four times and incubated with increasing concentrations of GST-REST (0-2 μg/mL). To measure binding levels, the wells were incubated for 2 h at room temperature with mouse monoclonal anti-GST antibody (Santa Cruz) diluted to 1% BSA in TBS. The wells were washed with TBST and incubated for 2 h at room temperature with 1% BSA in TBS containing horseradish peroxidase (HRP)-conjugated secondary antibody. After washing with TBST, 100 μL 3,3',5,5'-tetramethylbenzidine (TMB) solutions were added. After incubation for 20 min at room temperature, the reaction was stopped by addition of 100 μL of 1 N HCl.

For competitive ELISA experiments, PAH1 or PAH2 coated wells were pre-incubated with SAP25 or Mad1 peptide (0-100 μM) in TBST for 2 h at room temperature. After washing with TBST, GST-REST (2 μg/mL) was added to the wells and then incubated for 2 h. The bound GST-REST to PAH1 or PAH2 was detected by using anti-GST antibody as described above.

Cell permeability of peptide

P19 cells were treated with 2 μM of FITC-TAT-NLS-Mad1 peptide in culture media. The peptide (2 μM) containing culture media were replenished every 2 h until 24 h. After washing with PBS twice, the cells were fixed with 2.5% formaldehyde. Cell images were obtained by using confocal fluorescence microscopy (Zeiss, Germany).

Co-immunoprecipitation

P19 cells were lysed with cell lysis buffer (150 mM NaCl, 25 mM Tris-HCl, pH 7.4, 5% glycerol, 1% NP-40 and a protease inhibitor cocktail) for 10 min at 4 °C. After centrifugation at 15,000 rpm for 10 min at 4 °C, the cell lysates were pre-incubated with 20 μL of Protein G PLUS-agarose (Santa Cruz) in cell lysis buffer for 30 min at 4 °C. The pre-cleared cell lysates were incubated with SAP25 or Mad1 peptide (0, 30, 60 μM) for 2 h at 4 °C. Anti-REST or anti-Sin3 antibody was added to the cell lysates and incubated for 2 h at 4 °C. The lysates were incubated with Protein G PLUS-agarose on the rocker at 4 °C overnight. Immunoprecipitates were collected by centrifugation at 2,500 rpm for 5 min at 4 °C. After

washing with cell lysis buffer, the supernatant was discarded and pellets were resuspended with 40 μL of 2X electrophoresis sample buffer. Amounts of immunoprecipitates and co-precipitated proteins were analyzed by using western blot analysis.

Statistical Analysis

All data are reported as the mean ± s.d. from at least three independent experiments.

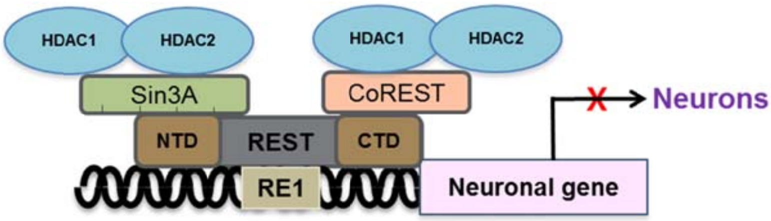

Figure S1. REST is a transcriptional factor that represses transcription of a number of neuronal genes. REST binds to a ~23 bp element termed the RE1 site (Repressor Element-1) via a DNA binding domain consisting of a cluster of 8 zinc fingers. Upon binding to the RE1 site, REST recruits its two corepressors, Sin3A and CoREST. CoREST interacts with the C-terminal domain of REST (amino acids 525-1097) while the N-terminal domain amino acids 1-152) of REST associates with Sin3A. Upon binding to REST, Sin3A recruits additional silencing factors, such as HDAC1/2 to facilitate the REST-mediated repressive mechanism in non-neuronal cells. On the other hand, Co-REST bound to the C-terminal domain of REST also recruits epigenetic enzymes HDAC1/2. Both Sin3A and CoREST exert distinct but overlapping biological roles which influence the function of its master regulator REST to restrict the neuronal phenotypes in non-neuronal cells. NTD: The N-Terminal Domain, CTD: The C-Terminal Domain.

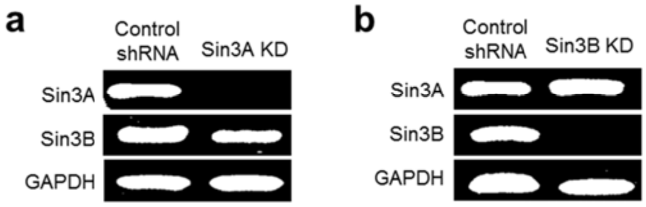

Figure S2. ShRNA mediated knockdown of Sin3A and Sin3B in P19 cells. P19 cells were stably transfected with each of Sin3A and Sin3B shRNA plasmids, and expression levels of (**a**) Sin3A and (**b**) Sin3B were then determined by using RT-PCR analysis. Scrambled shRNA (control shRNA) was used as a negative control and GAPDH as a loading control. All experiments were conducted at least three times.

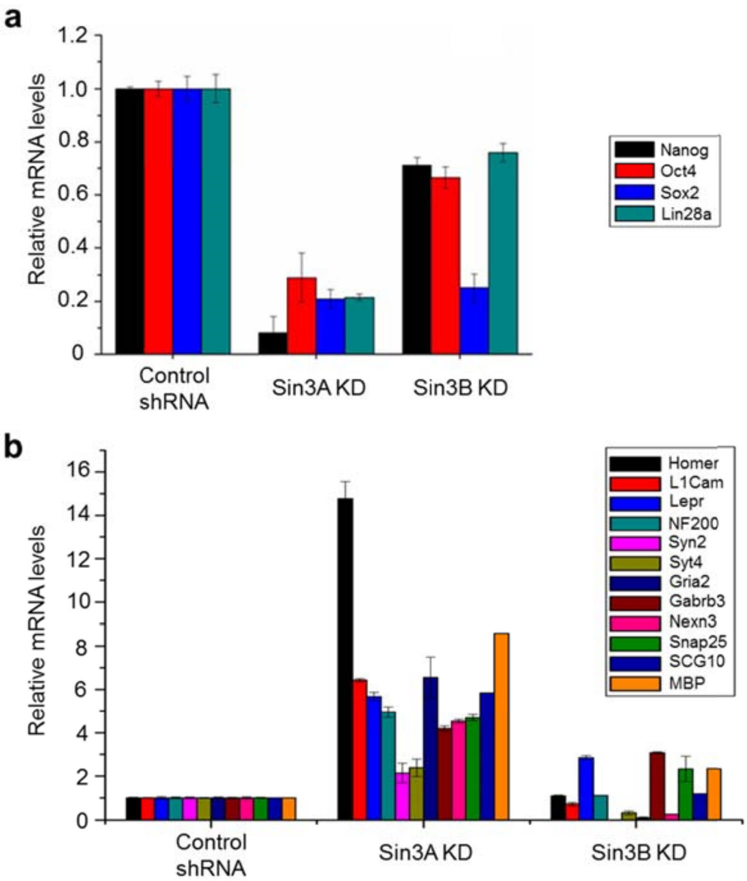

Figure S3. Effect of Sin3A and Sin3B knockdown on expression of REST target genes in P19 cells. Sin3A- and Sin3B-knockdown P19 cells were allowed to aggregate for 3 days and aggregated embryonic bodies were then dissociated into single cells. Sin3A- and Sin3B-knockdown cells were cultured in monolayers for 10 days. Transcriptional levels of REST target genes related to (**a**) stemness and (**b**) neurogenesis were determined by using quantitative RT-PCR analysis. Scrambled shRNA was used as a negative control (mean ± s.d., n = 3).

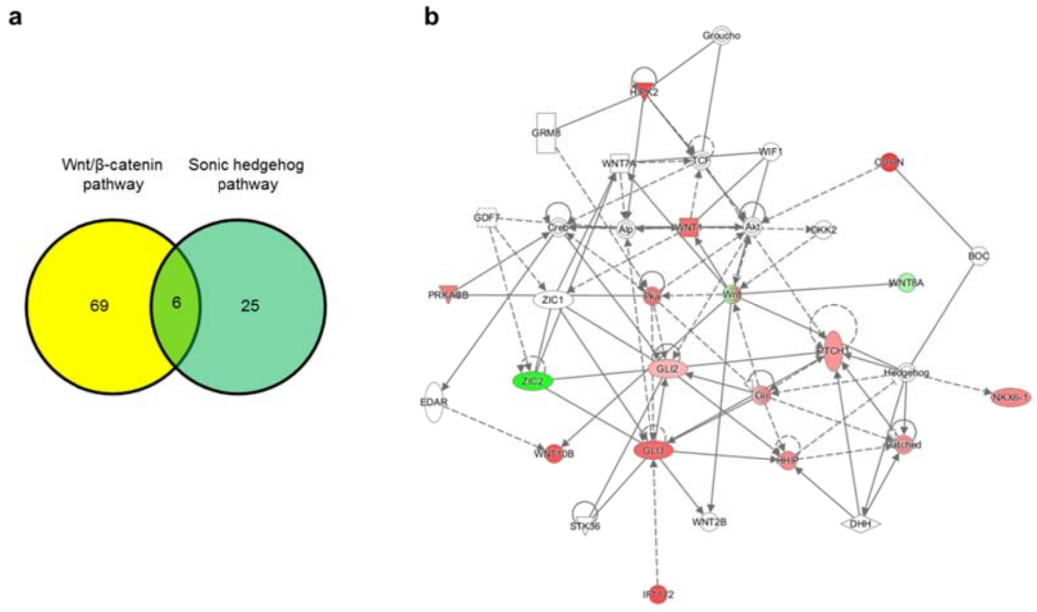

Figure S4. Transcriptional levels of genes associated with Wnt/β-catenin and Shh pathways are changed significantly in Sin3A-silenced P19 cells. (**a**) Venn diagram depicting Wnt/β-catenin and Shh pathways. The diagram shows the number of genes whose expression is significantly changed after 10 day incubation of Sin3A-silenced P19 cells. (**b**) Molecular relevant gene network showing a relationship between Wnt and Shh signaling. The network is displayed as nodes (gene and gene products) and edges (lines: biological relationships between nodes). Solid lines denote direct interactions, while dotted lines represent indirect interactions between genes presented in this network. The intensity of node color indicates the degree of (red) upregulation and (green) downregulation. Wnt pathway related genes (Wnt1, Wnt7a, Wnt10b, Hpk2 and Prkcab) were upregulated but a transcription regulator Zic2, which is known to inhibit the transcriptional activity of β-catenin, was downregulated in Sin3A knockdown cells. Shh pathway related genes (Ptch1, Gli2, Gli3, Wnt10b, Nkx6-1, Hhip and Cdon) were upregulated. Wnt1, Wnt2b, Wnt10b, and Prkcab are crosslinked with Wnt and Shh signaling pathways.

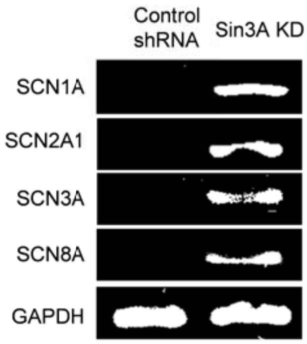

Figure S5. Expression of Voltage-gated sodium channel transcripts are expressed in Sin3A silenced P19 cells. Sin3A silenced P19 cells were allowed to aggregate in suspension for 3 days, and then the resulting aggregates were cultured in monolayer for 10 days. Transcriptional levels of several sodium channels were determined in differentiated cells by using RT-PCR analysis.

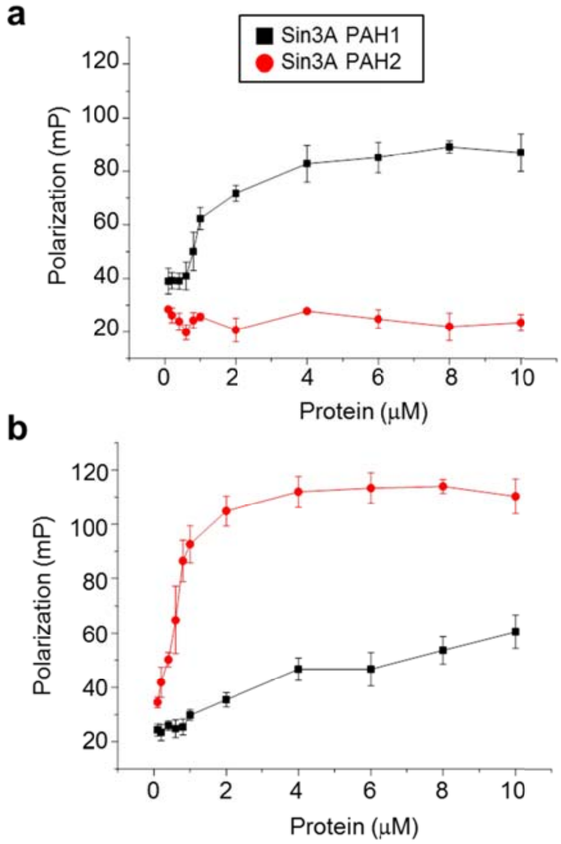

Figure S6. Binding of peptides to Sin3A PAH1 and PAH2 using fluorescence polarization. Various concentrations of Sin3A PAH1 or PAH2 were incubated with (**a**) 1 μM FITC-labeled SAP25 and (**b**) Mad1 peptides for 0.5 h. Fluorescence polarization is plotted as a function of protein concentrations (mean ± s.d., n = 3).

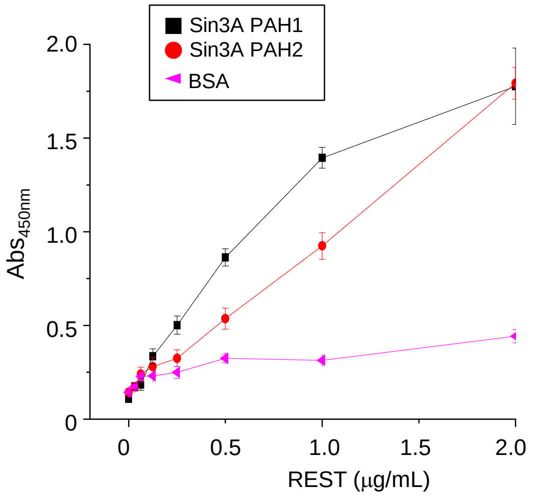

Figure S7. Binding of REST to Sin3A PAH1 and PAH2 domains using ELISA. The wells of microtiter plate were coated with Sin3A PAH1 or PAH2 (1 μg/mL). The coated wells were incubated with increasing concentrations of GST-REST for 2 h. Binding of REST to Sin3A PAH1 or PAH2 was measured by using anti-GST antibody (primary antibody) and HRP-conjugated secondary antibody (mean ± s.d., n = 3). BSA was used as a negative control.

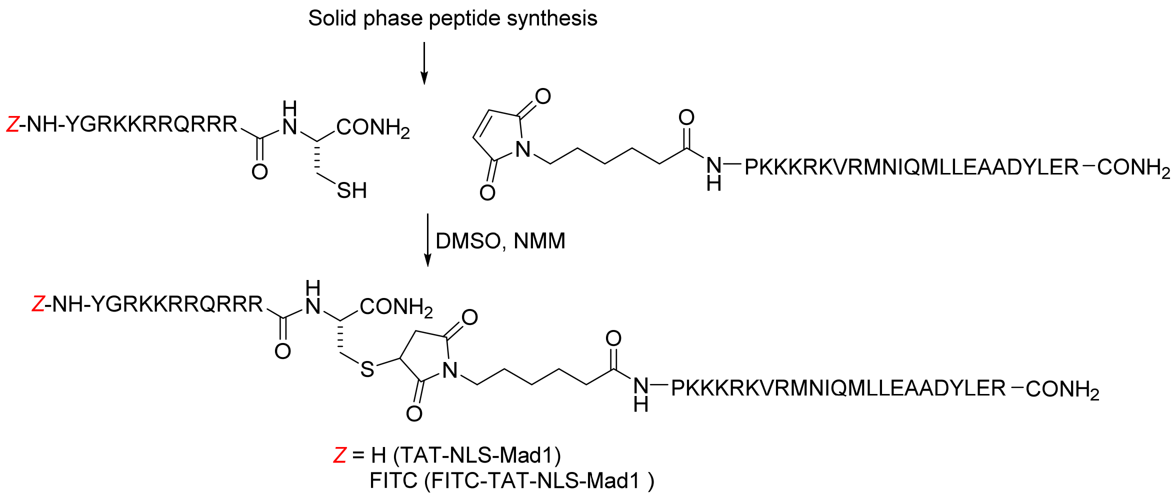

Figure S8. Synthesis of TAT-NLS-Mad1 peptide and its FITC-labeled form.

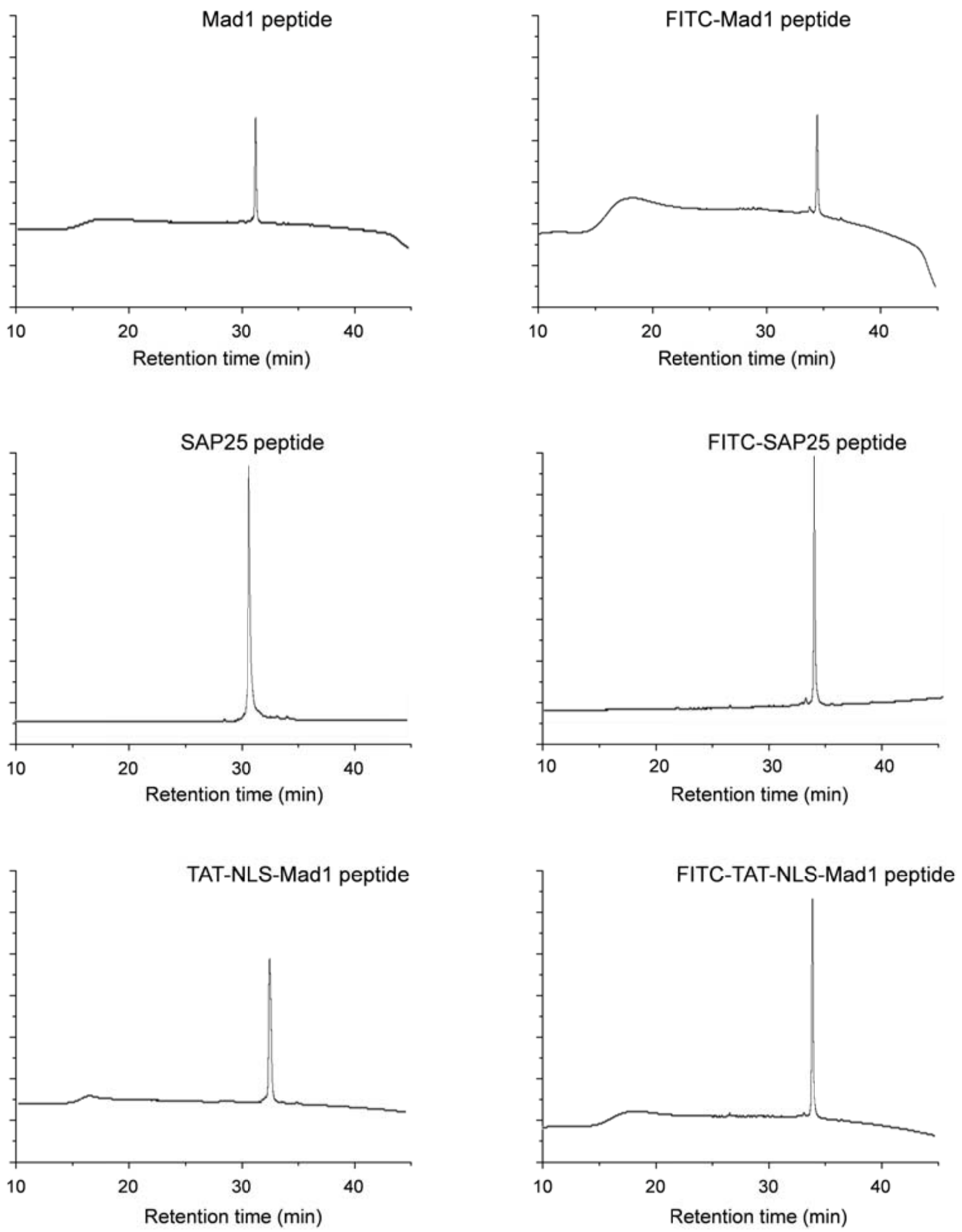

Figure S9. HPLC profiles of purified peptides.

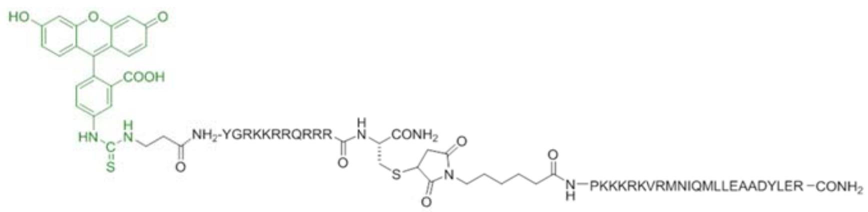

FITC-labeled TAT-NLS-Mad1

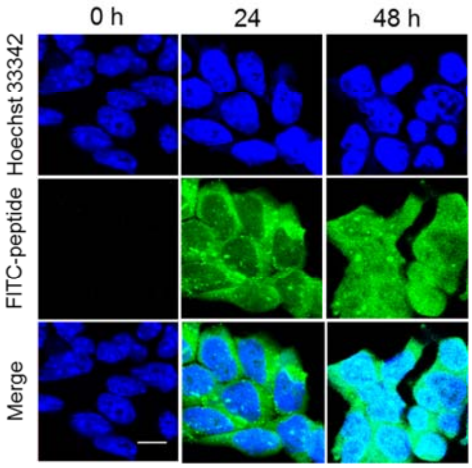

Figure S10. P19 cells were incubated with FITC-labeled TAT-NLS-Mad1 peptide during different time periods. Cell images were obtained by using confocal fluorescence microscopy (scale bar: 10 μm). Shown is a structure of FITC-labeled TAT-NLS-Mad1 peptide.

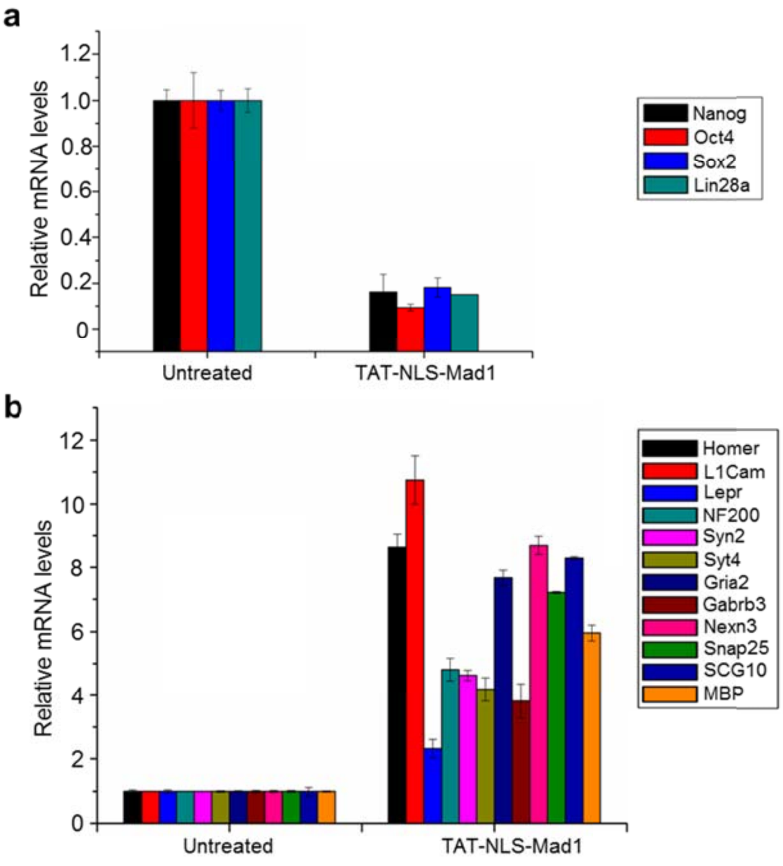

Figure S11. Effect of TAT-NLS-Mad1 on transcriptional levels of REST target genes in P19 cells. P19 cells were grown in suspension in the presence or absence of 30 μM TAT-NLS-Mad1 for a span of 3 days followed by monolayer culture for an additional 10 days. (**a**) Transcriptional levels of REST target genes related to stemness and (**b**) neurogenesis were determined by using quantitative RT-PCR analysis. 'Untreated' indicates no treatment of P19 cells with a peptide (mean ± s.d., n = 3).

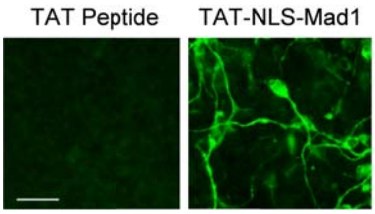

Figure S12. TAT peptide does not affect neuronal differentiation of P19 cells. P19 cells were incubated with 30 μM TAT or TAT-NLS-Mad1 peptide for 10 days. The cells were immunostained with Tuj1 antibody (bar: 50 μm).

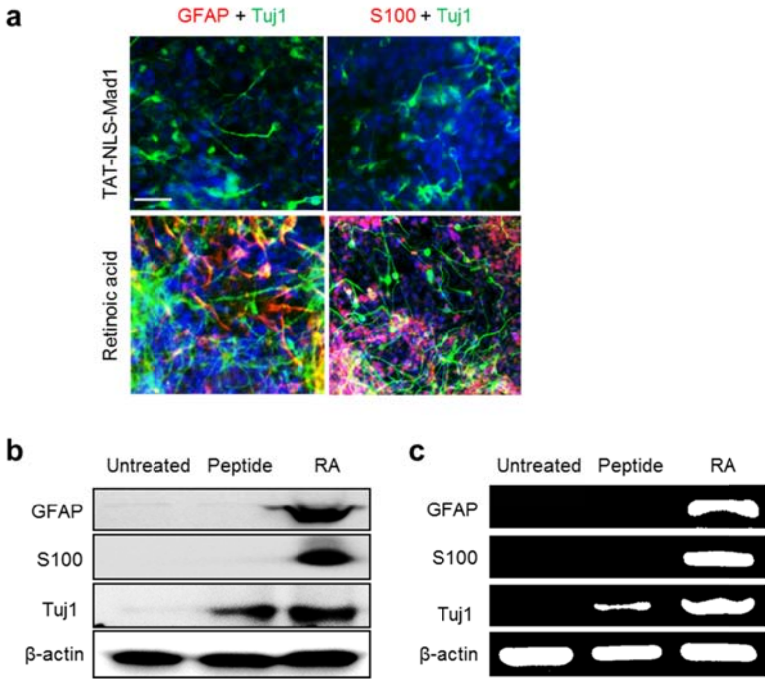

Figure S13. TAT-NLS-Mad1 peptide does not promote differentiation of P19 cells to astrocytes. P19 cells were grown in suspension in the presence or absence of 30 μM TAT-NLS-Mad1 for a span of 3 days followed by monolayer culture for 10 days. (**a**) The cells were immunostained with antibodies against neuronal (Tuj1, green) and astrocyte markers (GFAP and S100, red). Retinoic acid (1 μM) was used as a positive control for astrogenesis. The nucleus of cells was stained with DAPI (blue). Scale bar: 50 μm. (**b**) Expression of astrocyte markers in the peptide treated P19 cells was examined by using western blot and (**c**) RT-PCR analyses. 'Un' indicates no treatment of P19 cells with the peptide. All experiments were conducted at least three times.
